# Nutrient-derived signals regulate eosinophil adaptation to the small intestine

**DOI:** 10.1073/pnas.2316446121

**Published:** 2024-01-25

**Authors:** Vassily I. Kutyavin, Lisa L. Korn, Ruslan Medzhitov

**Affiliations:** ^a^Department of Immunobiology, Yale University School of Medicine, New Haven, CT 06510; ^b^Department of Medicine (Rheumatology), Yale University School of Medicine, New Haven, CT 06510; ^c^HHMI, Yale University School of Medicine, New Haven, CT 06510

**Keywords:** eosinophils, small intestine, retinoic acid, inflammation

## Abstract

Eosinophils are well recognized as effector cells of type 2 immunity, yet they also accumulate in many tissues under homeostatic conditions. However, the processes that govern homeostatic eosinophil accumulation and tissue-specific adaptation, and their functional significance, remain poorly defined. Here, we investigated how eosinophils adapt to the small intestine (SI) microenvironment and the local signals that regulate this process. We observed that eosinophils gradually migrate along the crypt–villus axis, giving rise to a villus-resident subpopulation with a distinct transcriptional signature. Retinoic acid signaling was specifically required for maintenance of this subpopulation, while IL-5 was largely dispensable outside of its canonical role in eosinophil production. Surprisingly, we found that a high-protein diet suppressed the accumulation of villus-resident eosinophils. Purified amino acids were sufficient for this effect, which was a consequence of accelerated eosinophil turnover within the tissue microenvironment and was not due to altered development in the bone marrow. Our study provides insight into the process of eosinophil adaptation to the SI, highlighting its reliance on nutrient-derived signals.

## Significance

In addition to protecting the host from pathogenic microbes, immune cells perform noncanonical physiological functions which require tissue-specific adaptation. Eosinophils are immune cells that are particularly abundant in the nutrient-rich small intestine (SI), but whether nutrients guide their adaptation to this tissue is largely unexplored. In this study, we found that eosinophils gradually migrate along the crypt–villus axis and develop into a transcriptionally distinct villus-resident subpopulation. We determined that this adaptation depends on retinoic acid, a metabolite of vitamin A. Furthermore, we unexpectedly found that high levels of dietary amino acids limit the accumulation of villus-resident eosinophils by accelerating eosinophil turnover. This study highlights the important role of dietary nutrients in shaping the immune cell populations of the SI.

Apart from their canonical functions in antimicrobial defense, many immune cells perform a wide range of homeostatic functions that protect tissue integrity and support physiologic processes (1–8). Homeostatic functions of immune cells depend on the tissues in which they reside, and circulating cells acquire tissue-specific characteristics upon recruitment that are either imparted through local signals or preprogrammed during development (9). These cell populations are regulated by distinct sets of signals that are immune cell lineage-specific. Notably, myeloid cells exhibit significant heterogeneity and can be further divided into circulating and tissue-resident subsets possessing unique tissue-specific characteristics (2, 6).

While eosinophils are generally regarded as effector cells that accumulate in the settings of type 2 inflammation, drug reactions, hypereosinophilic syndromes, and gastrointestinal diseases (10–13), they also appear in diverse forms under homeostatic conditions (14, 15). Like other myeloid cells, eosinophils can be found in circulating and tissue-resident pools. After eosinophil maturation in the bone marrow and entry into the circulation, processes that depend on the cytokine IL-5, eosinophils spend a short time in the circulation (half-life of only a few hours) before migrating into tissues or dying (10, 16–18). Several homeostatic functions of tissue eosinophils have been shown, including roles in tissue repair, glucose metabolism, and mammary gland development (19–23). The full range of eosinophil homeostatic functions and their underlying mechanisms are not well known.

Among all tissues, the small intestine (SI) contains the largest population of resident eosinophils in mice and humans under homeostatic conditions, owing to local signals that drive constitutive recruitment and extend survival of eosinophils (11, 17, 24, 25). Eosinophils in the SI have been shown to regulate local immune responses to microbes, parasites, and allergens and to influence tissue architecture in response to damage or microbial perturbation (14, 26–34). Several functions of SI eosinophils additionally depend on the presence and composition of the commensal microbiota (26, 33, 34). Eosinophil recruitment to the SI depends on the eotaxin chemokines, Ccl11 and Ccl24 (35, 36), while regulation of eosinophil survival is not fully understood; multiple factors, including IL-5, common gamma chain cytokines, SIRPα, IL-33, Neuromedin U, and retinoic acid (RA), have been implicated (17, 33, 34, 37–39).

Previous studies have shown that SI eosinophils are transcriptionally distinct from eosinophils in other tissues and consist of distinct subsets, although this heterogeneity remains poorly understood (39–41). In addition, recent studies have started to identify specific signaling pathways that regulate eosinophil adaptation to the SI (40–44). For example, two recent studies identified the aryl hydrocarbon receptor (AHR), a sensor of diverse environmental and endogenous molecules, as a regulator of eosinophil adaptation to the SI (41, 42). Still, the understanding of this process remains incomplete. In particular, it remains unclear whether the eosinophil heterogeneity and adaptation in the SI is related to their localization within the crypt–villus architecture, which contains zones with distinct epithelial cell composition and gene expression (45, 46). As eosinophils enter and migrate through these zones, the course of their adaptation might depend on signals that are produced in a zone-specific manner.

Intriguingly, eosinophils are most abundant in the proximal SI (comprising the duodenum and jejunum), the segment of the intestine most exposed to dietary nutrients, raising the question of whether these cells are regulated by nutrient-derived signals. Circulating eosinophil levels are known to depend on food intake by a mechanism involving type 2 innate lymphoid cell (ILC2)-derived IL-5 (18, 47–49), but outside of a few studies that examined the effects of high fat diet (41, 50), the impact of dietary nutrients on eosinophils within the SI itself remains largely unexplored.

Here, we show that eosinophils adapt to the SI under homeostatic conditions as they migrate up the crypt–villus axis, resulting in a villus-resident eosinophil subset with a distinct transcriptional signature. This adaptation depends on RA receptor (RAR) signaling and is suppressed by high levels of dietary amino acids, which accelerate eosinophil turnover in the tissue. Overall, we identified a homeostatic process of eosinophil adaptation to the SI that is regulated by nutrient-derived signals.

## Results

**Fig. 1.**
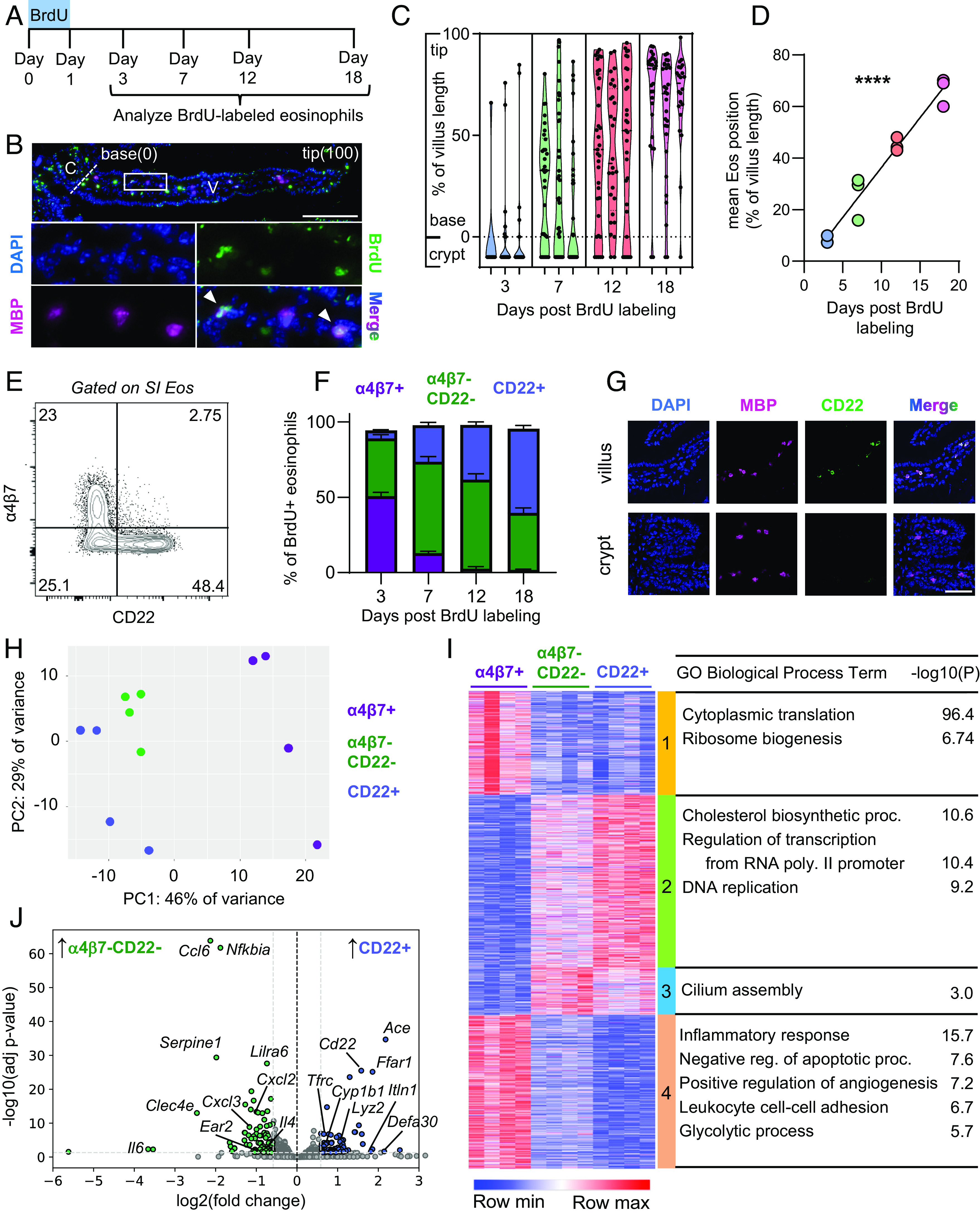
Eosinophil adaptation to the SI produces a distinct villus-resident subpopulation. (*A*) Diagram of BrdU pulse–chase protocol, showing the 24-h BrdU administration period followed by analysis of BrdU-labeled eosinophils in the proximal SI at indicated time points. (*B*) *Top*: Representative microscopy image of BrdU (green), MBP (magenta), and DAPI (blue) immunofluorescence staining in the crypt and villus of the proximal SI on day 12 of the experimental protocol shown in (*A*). (Scale bar, 100 μm.) Locations of the villus (V) tip, villus base, and crypt (C) are indicated. *Bottom*: Enlarged images of the outlined region in the top image showing the indicated staining. Arrowheads in the merged image indicate BrdU-labeled eosinophils (BrdU+ MBP+). (*C* and *D*) Violin plots (*C*) depict spatial distribution of BrdU-labeled eosinophils along the crypt–villus axis at different time points. Each violin plot represents an individual mouse, and each point represents an individual eosinophil. Position on the *y*-axis corresponds to the relative position along the length of the crypt–villus axis, based on the tissue landmarks depicted in (*B*) (100 = villus tip; 0 = villus base; <0 = crypt). The scatter plot (*D*) shows mean eosinophil positions calculated from the data in (*C*) (each point represents an individual mouse). Data are representative of 2 experiments with 3 mice per group. Data were analyzed by linear regression analysis (slope = 3.868, R^2^ = 0.96, ^∗∗∗∗^*P* < 0.0001). (*E*) Representative flow cytometry plot showing α4β7 and CD22 staining on proximal SI lamina propria (LP) eosinophils. (*F*) Quantification of α4β7+, α4β7− CD22−, and CD22+ subset frequencies in BrdU-labeled eosinophils isolated at indicated time points of the experimental protocol shown in (*A*) (representative of 2 experiments with 3 mice per group). Data in (*F*) are mean ± SEM. (*G*) Representative confocal immunofluorescence microscopy images depicting MBP (magenta), CD22 (green), DAPI (blue), and merged staining in crypt and villus regions of the proximal SI [representative of 2 experiments with 2 to 3 mice per group (Scale bar, 50 μm.)]. (*H*) Principal component analysis (PCA) of transcriptomes of α4β7+, α4β7− CD22−, and CD22+ eosinophil subsets isolated by FACS from the proximal SI LP. Each point indicates an individual biological replicate (2 mice pooled/replicate). (*I*) Heatmap of K-means (K = 4) clustering of transcriptomic profiles for differentially expressed genes (fold change > 1.5, adjusted *P*-value < 0.05) across the three eosinophil subsets. Each cluster is indicated by a different vertical color bar. Representative pathways (GO biological process) enriched in each cluster and their corresponding *P*-values are shown in the table on the *Right*. (*J*) Volcano plot depicting differentially expressed genes in a direct comparison of α4β7− CD22− and CD22+ SI eosinophils, plotted according to fold change and adjusted *P*-value. Genes enriched in α4β7− CD22− eosinophils are located to the left of the divider, while genes enriched in CD22+ eosinophils are located to the right.

To study how eosinophils adapt to the SI, we first used a BrdU pulse–chase approach to examine how their localization within the tissue microenvironment changes over the course of their residency. In this method, proliferating eosinophil progenitors are temporarily labeled with BrdU during a 24-h pulse, giving rise to a limited population of mature eosinophils that retain the BrdU label indefinitely because they are postmitotic. Using immunofluorescence microscopy, we identified this population of BrdU+ eosinophils at different time points after the initial labeling, allowing us to track their location in the tissue over time (Fig. 1 *A* and *B*). We focused on the proximal SI (duodenum and jejunum) because it contains the majority of SI eosinophils (33). At the earliest time point (3 d after BrdU), BrdU+ eosinophils were concentrated primarily around the crypts, with few cells identified in the villi (Fig. 1 *C* and *D*). Over the next 15 d (the extent of eosinophil survival) (17), the distribution of eosinophils gradually shifted toward the tips of the villi (Fig. 1 *C* and *D*). These data indicate that eosinophils initially enter the tissue near the crypts and subsequently migrate along the crypt–villus axis, resulting in the older cells being concentrated in the upper villus.

We next asked whether eosinophil residence in the SI was associated with changes to cellular phenotype. Initial analysis of known eosinophil surface molecules by flow cytometry revealed three subsets distinguished by variable expression of the gut-homing integrin α4β7 and the lectin CD22 (α4β7+, α4β7− CD22−, and CD22+ subsets) (Fig. S1*E* and *SI Appendix*, Fig. S1*A*). CD22 was previously shown to be specifically expressed on eosinophils in the SI (44), suggesting that it marks a unique population adapted to this tissue environment. Combining our BrdU pulse–chase approach with flow cytometry, we found that expression of α4β7 and CD22 was highly correlated with eosinophil age: α4β7 marked younger eosinophils, while CD22 was up-regulated on a subset of eosinophils over time, and an intermediate population expressed neither marker (Fig. 1*F* and *SI Appendix*, Fig. S1*B*). Consistent with our earlier observation of older eosinophils localizing to the villi (Fig. 1*C*), we found that CD22 was selectively expressed by a subset of villus-resident eosinophils (Fig. 1*G*). The three populations also differed in their size and total granularity (*SI Appendix*, Fig. S1*C*), as well as the expression of CD11c and PD-L1 (*SI Appendix*, Fig. S1*D*), two other surface markers previously shown to distinguish subsets of intestinal eosinophils (40, 41, 43). These data indicate that eosinophils undergo additional adaptation as they migrate from crypt to villus.

To investigate the transcriptional changes occurring during eosinophil adaptation to the SI, we sorted α4β7+, α4β7− CD22−, and CD22+ eosinophils (corresponding approximately to younger, intermediate, and older eosinophils, respectively) and subjected them to RNA sequencing (29). The three populations exhibited distinct transcriptional profiles (Fig. 1*H*). K-means clustering followed by gene ontology analysis identified several pathways enriched among the differentially expressed genes (Fig. 1*I*). For example, the α4β7− CD22− and CD22+ subsets exhibited lower expression of genes involved in translation and glycolysis, and higher expression of genes involved in cholesterol synthesis (Fig. 1*I* and *SI Appendix*, Fig. S1*E*), indicating that eosinophils undergo metabolic reprogramming during their adaptation to the SI. In addition, these subsets displayed lower expression of many genes involved in inflammation and higher expression of numerous transcription factors (Fig. 1*I* and *SI Appendix*, Fig. S1*F*), suggesting that tissue adaptation might reduce inflammatory potential in favor of alternative programs that operate during homeostasis. Further comparison of the CD22+ and α4β7− CD22− subsets revealed differential expression of many genes involved in innate immunity, including those encoding cytokines (*Il4* and *Il6*), chemokines (*Ccl6, Cxcl2,* and *Cxcl3*), pattern recognition molecules (*Clec4e* and *Itln1)*, and antimicrobial products (*Lyz2*, *Defa30,* and *Ear2*) (Fig. 1*J*). CD22+ cells also exhibited higher expression of several genes involved in diverse aspects of intestinal homeostasis, including *Ace*, *Tfrc,* and *Cyp1b1* (51–53). Overall, these transcriptional data reveal a wide range of functional adaptations occurring in eosinophils during their residency in the SI.

Next, we investigated the homeostatic functions of SI eosinophils by comparing eosinophil-deficient ΔdblGATA mice to their wild-type (WT) littermates (54). We observed a modest but significant decrease of villus area in ΔdblGATA mice, indicating that eosinophils contribute to regulation of villus architecture (*SI Appendix*, Fig. S2 *A*–*C*), consistent with a recent study (33). This was not accompanied by altered enterocyte zonation, as indicated by unchanged expression of c-Maf-dependent genes (*SI Appendix*, Fig. S2*E*) (46). In contrast to earlier reports (33, 34), we did not observe altered epithelial migration, gut motility, or intestinal permeability in ΔdblGATA mice (*SI Appendix*, Fig. S2 *D*, *F*, and *G*). This discrepancy may be due to differences in microbiota composition in mice from different facilities since eosinophil function can be influenced by intestinal microbes (33, 34). In addition, flow cytometric analysis of SI lamina propria (LP) revealed reductions of multiple other immune cell populations in ΔdblGATA mice, including monocytes, dendritic cells, ILC2s, and several T cell subsets, suggesting a role for eosinophils in the recruitment or maintenance of these populations (*SI Appendix*, Fig. S2 *H*–*L*). Taken together, these data are consistent with eosinophil involvement in regulation of villus architecture and immune cell composition, while other reported functions may depend on the presence of specific microbial species that were absent in our mouse colony. As we recognized that the functional contributions of eosinophils in our mouse colony were relatively limited, we decided to focus instead on the regulation of eosinophil adaptation to the SI.

**Fig. 2.**
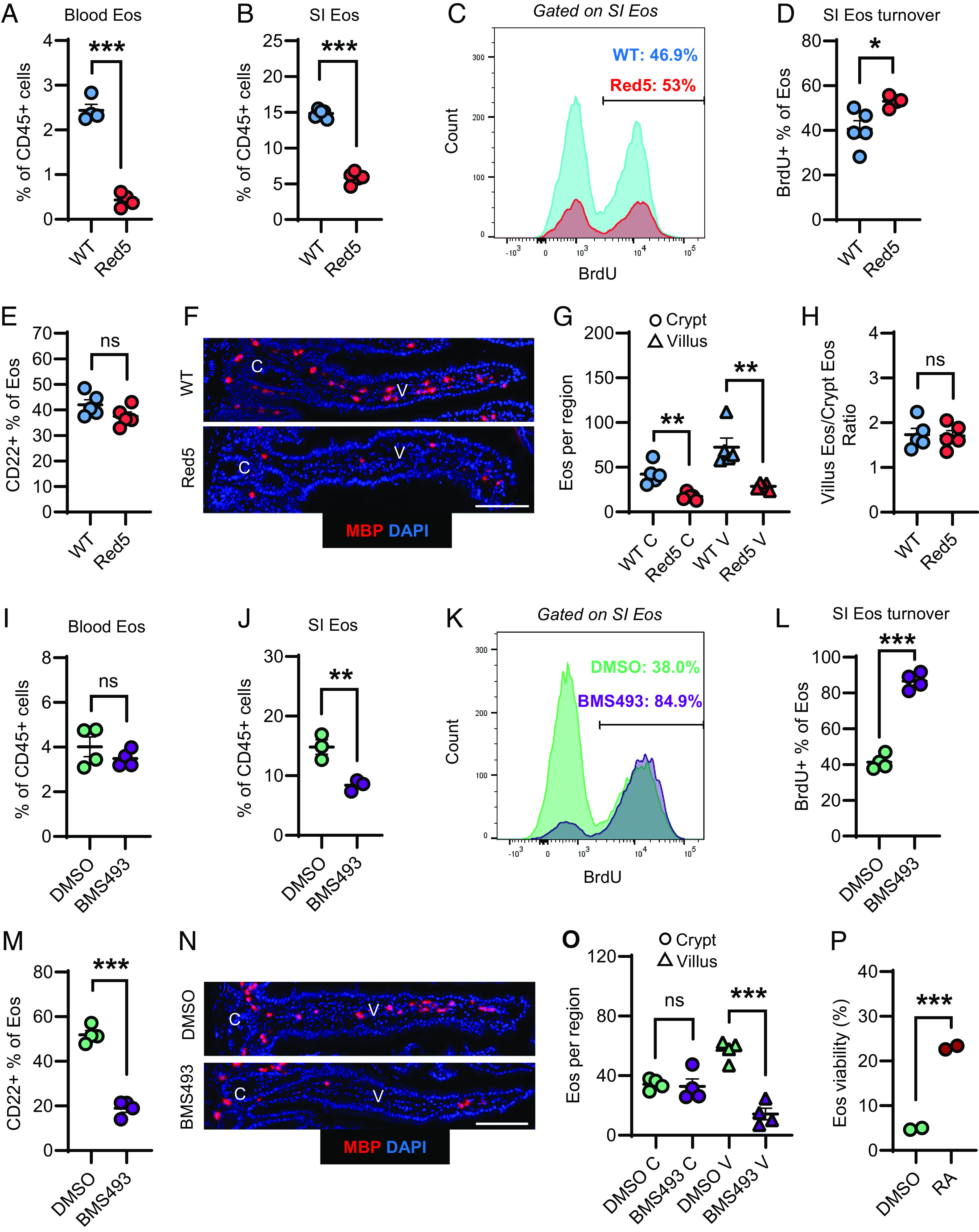
IL-5 regulates overall eosinophil accumulation, while RAR signaling is specifically required for maintenance of villus-resident CD22+ eosinophils. (*A* and *B*) Frequency of eosinophils (% of total live CD45+ cells) in blood (*A*) or SI LP (*B*) from WT or Red5 (homozygous) mice (representative of 2 experiments with 3 to 5 mice per group). (*C* and *D*) Representative flow cytometry analysis of eosinophil BrdU incorporation (*C*) and frequency of BrdU+ eosinophils (*D*) in the SI LP of WT or Red5 mice after 7 d of BrdU labeling (representative of 2 experiments with 4 to 5 mice per group). (*E*) Percent of eosinophils that were CD22+ (by flow cytometry) from the SI LP of WT or Red5 mice (representative of 3+ experiments with 3 to 5 mice per group). (*F*–*H*) Representative microscopy images of MBP (red) and DAPI (blue) immunofluorescence staining [*F*; (Scale bar, 100 μm.)] and corresponding quantification of eosinophils near crypts (C) or in villi (V), per region, from WT or Red5 mice (*G*). (*H*) The ratio of villus to crypt eosinophils for each genotype, calculated from data in (*G*). Representative of 2 experiments with 4 to 5 mice per group. (*I* and *J*) Frequency of eosinophils (% of total live CD45+ cells) in blood (*I*) or SI LP (*J*) from mice treated with DMSO or BMS493 for 8 d (representative of 2 and 3 experiments with 3 to 5 mice per group). (*K* and *L*) Representative flow cytometry analysis of eosinophil BrdU incorporation (*K*) and frequency of BrdU+ eosinophils (*L*) in SI LP of mice treated with DMSO or BMS493 for 8 d, including 7 d of BrdU labeling (representative of 2 experiments with 4 to 5 mice per group). (*M*) Percent of eosinophils that were CD22+ (by flow cytometry) from SI LP of mice treated with DMSO or BMS493 for 8 d (representative of 3 experiments with 3 to 5 mice per group). (*N* and *O*) Representative microscopy images of MBP (red) and DAPI (blue) immunofluorescence staining [*N*; (Scale bar, 100 μm.)] and corresponding quantification of eosinophils near crypts (C) or in villi (V), per region, from mice treated with DMSO or BMS493 for 8 d (*O*). Representative of 2 experiments with 4 to 5 mice per group. (*P*) Eosinophil viability after purified SI LP eosinophils were cultured with RA or DMSO (vehicle control) for 48 h. Representative of 2 experiments with 2 replicates per condition. Data are presented as mean ± SEM. All data were analyzed by Student’s *t* test. ^∗∗∗^*P* < 0.001, ^∗∗^*P* < 0.01, ^∗^*P* < 0.05, and ns = not significant (*P* > 0.05).

Our next goal was to identify the molecular signals that regulate eosinophil adaptation to the SI. In particular, we searched for signals that are required for the maintenance of CD22+ villus-resident eosinophils. We first tested the role of IL-5, which is generally considered to be the most important signal for eosinophil survival and differentiation (55). Consistent with earlier studies (35), IL-5-deficient Red5 mice exhibited a dramatic reduction of eosinophils in the blood and the SI (Fig. 2 *A* and *B*) (18). However, eosinophil turnover in the SI (based on BrdU labeling) was only modestly elevated in Red5 mice (Fig. 2 *C* and *D*). Furthermore, despite the overall eosinophil reduction, the relative frequency of CD22+ eosinophils (Fig. 2*E*) and the ratio of villus to crypt eosinophils (Fig. 2 *F*–*H*) were unchanged in Red5 mice. These data indicate that while IL-5 is important for overall production of eosinophils, and therefore determines how many reach the SI, it is largely dispensable for eosinophil adaptation within the SI microenvironment.

We then tested the roles of other candidate signals, including IL-33 and retinoid acid (RA), both of which are produced by multiple cell types in the SI and have been previously shown to modulate eosinophil survival (33, 34, 38). Genetic deficiency of the IL-33 receptor (*Il1rl1*^−/−^) did not significantly affect eosinophil abundance nor the frequency of CD22+ eosinophils in the SI (*SI Appendix*, Fig. S3 *A* and *B*), indicating that IL-33 signaling is not required for tissue adaptation in this context. To test the role of RA, we inhibited RARs by administering the inverse agonist BMS493 (34, 56). RAR inhibition resulted in a reduction of SI eosinophils, while eosinophil frequency in blood was unchanged (Fig. 2 *I* and *J*). Moreover, we found that inhibition of RARs dramatically accelerated SI eosinophil turnover (Fig. 2 *K* and *L*) and resulted in selective loss of CD22+ villus-resident eosinophils (Fig. 2 *M*–*O*). SI eosinophils expressed RARα and RARγ, suggestive of a direct mechanism of action (*SI Appendix*, Fig. S3*C*). To test whether RA can act directly on SI eosinophils to promote their survival, we purified SI eosinophils and cultured them with or without RA. Treatment with RA resulted in increased survival (Fig. 2*P*). Taken together, these data indicate that RA signaling via RARs positively regulates SI eosinophil survival and is required for the accumulation of villus-resident CD22+ eosinophils.

**Fig. 3.**
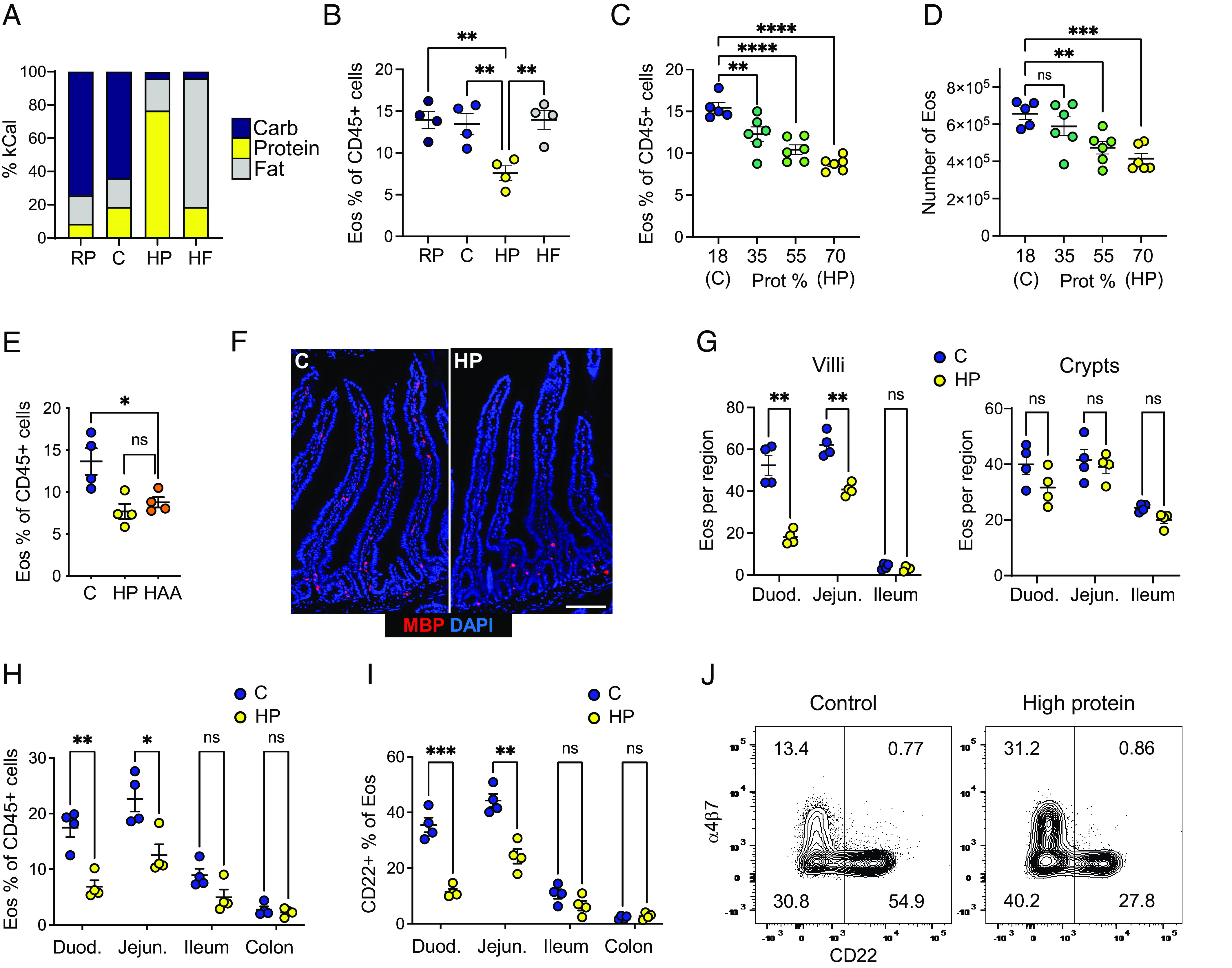
High-protein diet reduces villus-resident CD22+ eosinophils. (*A*) Percent of kilocalories (% kCal) from different macronutrients for diets shown in *B*. RP = reduced (8%) protein, C = control (18% protein), HP = high (70%) protein, and HF = high fat. (*B*) Eosinophil frequency in the proximal SI lamina propria (LP) of mice fed diets containing variable macronutrient composition for 15 d, representative of at least 3 experiments with 3 to 4 mice per group. (*C* and *D*) Eosinophil frequency (*C*) and number (*D*) in the proximal SI LP of mice fed diets containing the indicated percentage of protein (15 d), representative of 2 experiments with 4 to 6 mice per group. (*E*) Eosinophil frequency in proximal SI LP of mice fed either C, HP, or a diet containing elevated amino acids (“HAA”) in amounts equivalent to those in HP (15 d), representative of 2 experiments with 4 mice per group. (*F* and *G*) Representative microscopy images (*F*) and quantification (*G*) of eosinophils near crypts and in villi, per region, of the proximal SI of mice fed C or HP diets [red = MBP, blue = DAPI, (Scale bar, 100 μm.) “Duod.” = duodenum, “Jejun.” = jejunum]. (*H*) Eosinophil frequency in LP of the indicated regions of the gastrointestinal (GI) tract in mice fed C or HP diets for 15 d, representative of 2 experiments with 4 mice/group. (*I*) Percent of eosinophils that were CD22+ in LP of the indicated regions of the GI tract in mice fed C or HP diet for 15 d, representative of 2 experiments with 4 mice per group. (*J*) Representative flow cytometry plots showing α4β7 and CD22 staining in proximal SI LP eosinophils in mice fed C or HP diet for 15 d. Data are presented as mean ± SEM. All data were analyzed by Student’s *t* test. ^∗∗∗∗^*P* < 0.0001, ^∗∗∗^*P* < 0.001, ^∗∗^*P* < 0.01, ^∗^*P* < 0.05, and ns = not significant (*P* > 0.05).

Since RA is a nutrient-derived signal, and given the abundance of eosinophils in the nutrient-rich proximal SI, we asked more generally whether diet could influence eosinophil adaptation to this tissue. Based on earlier studies which showed reduction of blood eosinophils after overnight fasting (18, 47, 49), we decided to test the effects of overnight fasting, intermittent (every other day) fasting, or chronic food restriction (60% of ad libitum controls) on eosinophil accumulation in the SI. While we confirmed the reduction of blood eosinophils after overnight fasting (*SI Appendix*, Fig. S4*A*), none of these interventions affected the frequency of eosinophils in the SI (*SI Appendix*, Fig. S4 *A*–*C*). Next, we asked whether dietary macronutrient composition influences eosinophil adaptation to the SI. In consultation with a nutritionist, we designed diets with variable macronutrient compositions to isolate the effects of carbohydrates, protein, and fat, while keeping the levels of the other dietary components, including micronutrients, constant (Fig. 3*A*). Surprisingly, increasing dietary protein (to 70% by mass) led to a robust and selective reduction of eosinophil frequency in the SI (Fig. 3*B* and *SI Appendix*, Fig. S4*D*). In contrast, altering the amount of dietary carbohydrates or fat did not affect eosinophil frequency (Fig. 3*B*). We next asked whether the effect of high-protein feeding extended to eosinophils located in other tissues. Eosinophil frequency was not significantly altered in most other tissues, except the stomach and thymus, where eosinophils were also reduced (*SI Appendix*, Fig. S4*E*).

The eosinophil reduction in the SI was protein dose-dependent, with modest but significant reductions in eosinophil frequency and number observed at more moderate increases in dietary protein to 35% and 55% (Fig. 3 *C* and *D*). We further tested whether intact protein structure was required for the effect by administering a diet with an elevated amount of purified amino acids instead of intact protein. We found that feeding purified amino acids was sufficient to reduce SI eosinophils (Fig. 3*E*). Strikingly, the high-protein diet led to the specific depletion of the villus-resident eosinophil subset in the proximal SI, while crypt eosinophils were maintained (Fig. 3 *F* and *G*), similar to the effect of RAR inhibition (Fig. 2 *N* and *O*). Consistent with this finding, the high-protein diet caused a reduction of CD22+ eosinophils in the proximal SI, while the α4β7+ and α4β7-CD22- populations were proportionally increased (Fig. 3 *H*–*J*). Taken together, these data indicate that dietary macronutrient composition, specifically the amount of dietary amino acids, influences eosinophil adaptation to the SI.

**Fig. 4.**
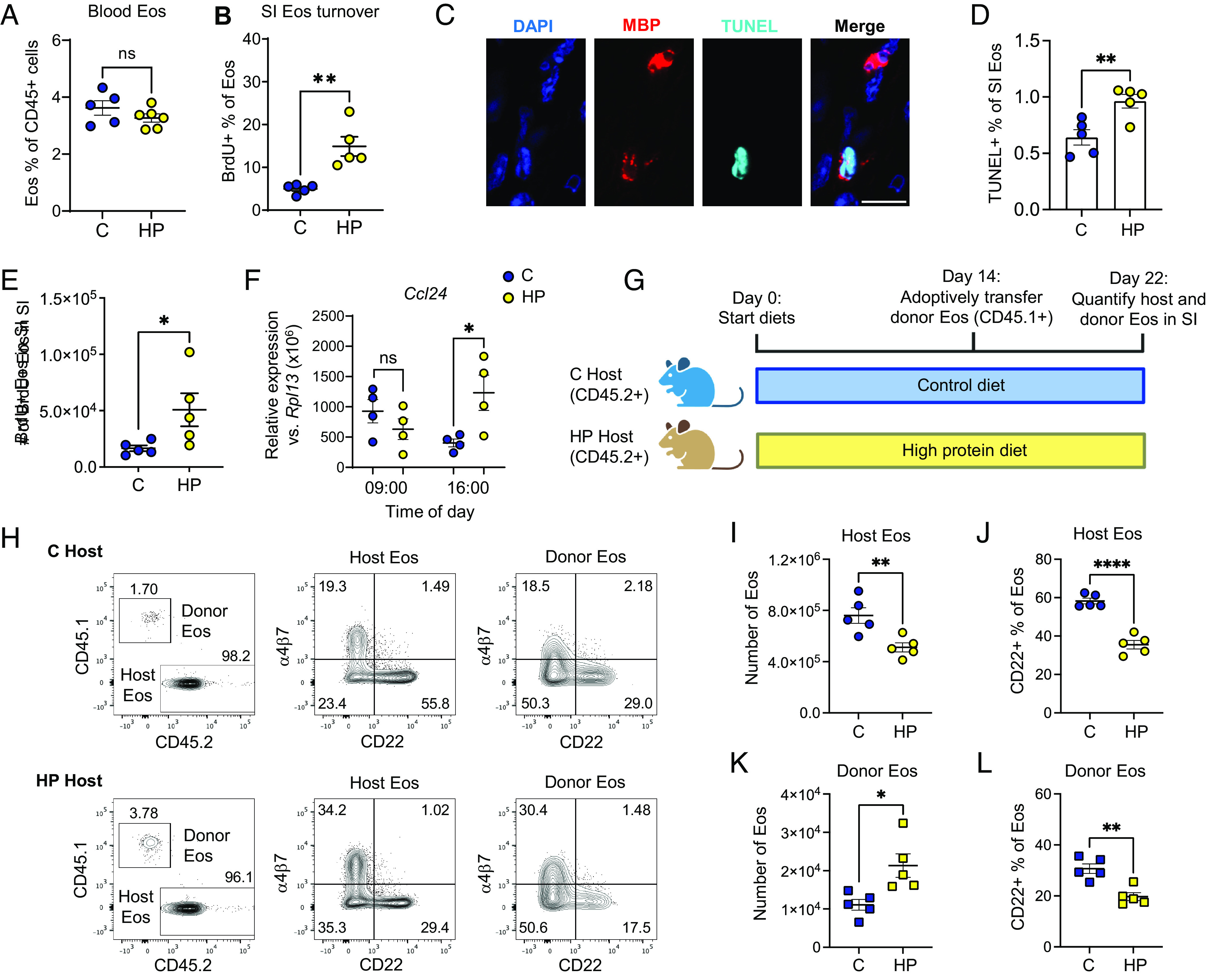
High-protein diet accelerates SI eosinophil turnover independently of alterations to eosinophil development. (*A*) Eosinophil frequency in blood from mice fed control (C) or high-protein (HP) diets for 15 d, representative of greater than 3 experiments with 3 to 6 mice per group. (*B*) Percent of proximal SI lamina propria (LP) eosinophils that were labeled by BrdU after 3 d of labeling, representative of 3 experiments with 3 to 5 mice per group. (*C*) Representative images of MBP, TUNEL, and DAPI immunofluorescence staining in the proximal SI. (Scale bar, 10 μm.) (*D*) Quantification of apoptosis (TUNEL+) in proximal SI eosinophils (MBP+) in mice fed C or HP diets for 15 d, representative of 3 experiments with 4 to 5 mice/group. (*E*) Absolute number of proximal SI LP eosinophils that were labeled by BrdU after 3 d of labeling during feeding with the indicated diets (days 12 to 15), representative of 3 experiments with 3 to 5 mice per group. (*F*) Eotaxin-2 (*Ccl24*) gene expression in the proximal SI epithelium after feeding with the indicated diets for 15 d and sample collection at the indicated times of day, representative of 3 experiments with n = 3 to 6 mice per group. (*G*–*L*) Eosinophil adoptive transfer experiment, representative of two experiments with 4 to 5 recipient mice per group: Schematic showing experimental design (*G*), representative flow cytometric analyses of host (CD45.2+) and donor (CD45.1+) eosinophils isolated from proximal SI LP of host (recipient) mice fed C or HP diets (*H*), number of host eosinophils in SI (*I*), percent of host eosinophils that were CD22+ (*J*), number of donor eosinophils in SI (*K*), and percent of donor eosinophils that were CD22+ (*L*) in recipient mice fed C or HP diet, on day 22 of the experimental protocol shown in G. Data are presented as mean ± SEM. All data were analyzed by Student’s *t* test. ^∗∗∗∗^*P* < 0.0001, ^∗∗∗^*P* < 0.001, ^∗∗^*P* < 0.01, ^∗^*P* < 0.05, and ns = not significant (*P* > 0.05).

The reduction of SI eosinophils in response to elevated dietary amino acids could be due to altered eosinophil production, recruitment, or lifespan in the tissue. High-protein diet did not alter the levels of circulating eosinophils, suggesting that eosinophil production was not suppressed (Fig. 4*A*). We next measured SI LP eosinophil turnover by BrdU labeling and found that turnover was substantially increased in mice fed the high-protein diet, indicating reduced eosinophil lifespan (Fig. 4*B*). In line with this finding, we detected a higher frequency of apoptotic eosinophils after high-protein diet feeding (Fig. 4 *C* and *D*). Isolated SI eosinophils did not exhibit greater cell death in response to elevated amino acid concentrations ex vivo compared to other LP immune cells, suggesting that our observations in vivo are unlikely to be a consequence of direct exposure to high amino acid concentration (*SI Appendix*, Fig. S5*A*). Taken together, these data indicate that the reduced lifespan of SI eosinophils in response to high-protein diet is at least in part due to increased apoptosis triggered indirectly by signals downstream of dietary amino acids.

Surprisingly, despite the overall reduction of SI eosinophils in response to high-protein diet, BrdU labeling also revealed an increase of recently recruited SI eosinophils (number of BrdU+ eosinophils in the SI after 3 d of labeling) in mice fed the high-protein diet compared to the control diet (Fig. 4*E*). To explore the mechanisms underlying this increased recruitment, we measured expression of genes encoding chemokines that are known to attract eosinophils to the SI. We found increased expression of eotaxin-2 (*Ccl24*) in the SI epithelium in the afternoon, suggesting a possible role for this chemokine in the increased eosinophil recruitment during high-protein feeding (Fig. 4*F*).

We also addressed other potential mechanisms that might operate downstream of dietary amino acids. We first tested the involvement of the microbiota, which are known to influence the functions of intestinal eosinophils and are sensitive to dietary macronutrient composition (26, 33). High-protein feeding still resulted in loss of CD22+ eosinophils in germfree mice and in mice treated with broad-spectrum antibiotics, indicating that the microbiota are not required for the dietary effect (*SI Appendix*, Fig. S5 *B*–*E*). In addition, we tested the role of AHR, a nuclear receptor that integrates microbial and dietary cues, which was recently found to regulate eosinophil adaptation to the SI (41, 42). Dietary supplementation of Indole-3-carbinol (I3C), an AHR ligand precursor (57), led to increased eosinophil accumulation without altering the proportion of CD22+ cells, suggesting that signaling through AHR does not mediate the effect of high-protein diet (*SI Appendix*, Fig. S5 *F* and *G*). Finally, since both high-protein diet feeding and inhibition of RA signaling resulted in accelerated eosinophil turnover and loss of villus-resident eosinophils, we asked whether these two signals act through the same or distinct pathways. We observed that high-protein diet still reduced eosinophil accumulation when RAR signaling was inhibited by BMS493 (*SI Appendix*, Fig. S5*H*). Furthermore, expression of RAR target genes (34, 58) was largely unchanged in SI of mice fed high-protein diet, suggesting that dietary amino acids and RA act through distinct pathways to regulate eosinophil adaptation to the SI (*SI Appendix*, Fig. S5 *H*–*J*).

Finally, to determine whether the effects of high-protein diet depended on alterations to signals imparted upstream during eosinophil development, we used an adoptive transfer approach (Fig. 4*G*). To obtain a large number of donor eosinophils, congenic (CD45.1+) mice fed normal chow were injected with IL-5 plasmid to boost endogenous eosinophil production (59). Splenic CD45.1+ eosinophils from IL-5-plasmid boosted mice were adoptively transferred into CD45.2+ mice that had been fed either control or high-protein diet for the preceding 2 wk. Eight days after transfer, donor (CD45.1+) and host (CD45.2+) eosinophils in the SI of recipient mice were analyzed (Fig. 4*H*). As expected, there were fewer host eosinophils and fewer CD22+ eosinophils in the mice fed the high-protein diet (Fig. 4 *I* and *J*). Consistent with increased eosinophil recruitment, more donor eosinophils were recovered from mice fed the high-protein diet (Fig. 4*K*). Importantly, donor eosinophils recovered from recipients fed high-protein diet still exhibited a reduced frequency of CD22+ cells compared to donor eosinophils recovered from recipients fed the control diet (Fig. 4*L*). Since the donor eosinophils developed in mice fed normal chow, yet still responded to high-protein diet after transfer, these results indicate that the effects of high-protein diet do not depend on alterations to eosinophil development in the bone marrow and instead are probably a consequence of altered signals produced in the SI.

## Discussion

**Fig. 5.**
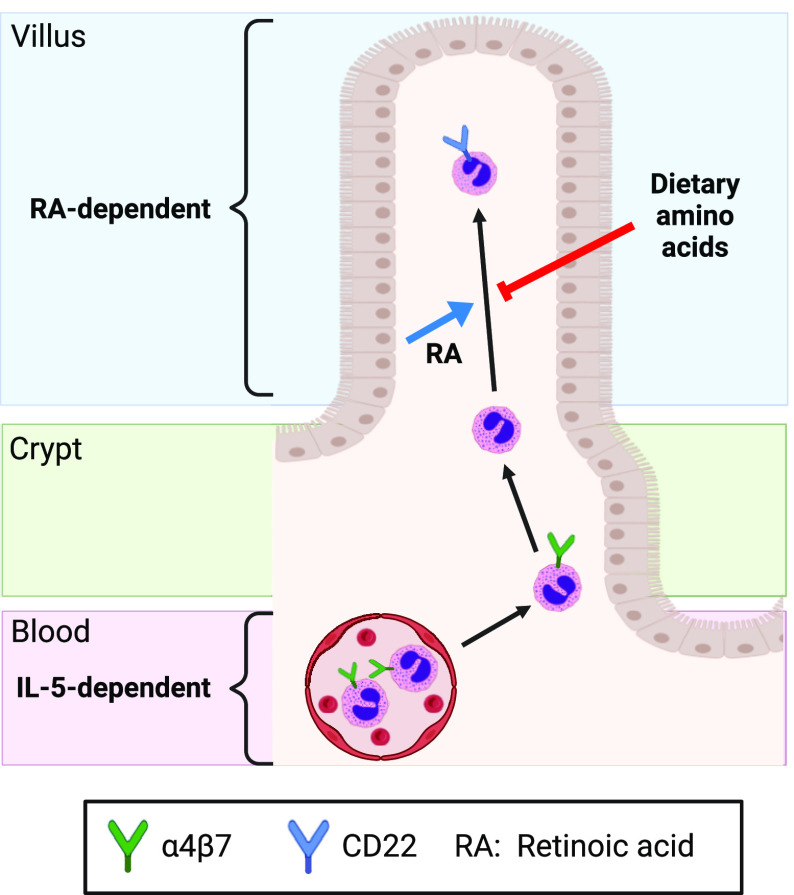
Model: regulation of eosinophil adaptation to the SI by nutrient-derived signals. Circulating eosinophils depend on IL-5 and enter the SI near the crypts. As eosinophils adapt to the tissue, they down-regulate α4β7, up-regulate CD22, migrate into the villi, and exhibit an altered transcriptional profile. Maintenance of CD22+ villus-resident eosinophils depends on RA signaling and is inhibited by high levels of dietary amino acids.

Recent studies demonstrated the existence of tissue-resident eosinophil subsets with distinct transcriptional profiles (14, 42), but little is known about the processes that guide the development of these specialized populations. Here, we report that eosinophil adaptation to the SI is characterized by gradual redistribution along the crypt–villus axis and transcriptional remodeling. Furthermore, we find that this adaptation is regulated by multiple nutrient-derived signals, including RA and dietary amino acids (Fig. 5). Our results, therefore, highlight the importance of the nutritional milieu as a key factor in the SI microenvironment that shapes eosinophil identity.

Our study builds on earlier phenotypic characterization of SI eosinophil subsets by demonstrating how aspects of the population heterogeneity are related to spatial and temporal dimensions of tissue residency. Newly recruited eosinophils that are concentrated near the crypts (α4β7+) exhibit a distinct surface phenotype and transcriptome compared to eosinophils that already migrated into the villi (CD22+). Whether this transition from crypt to villus is fixed or reversible, analogous to reversible macrophage polarization (2), remains an open question and could be addressed with fate mapping tools. It is likely that migration of eosinophils from crypt to villus exposes them to a different milieu of instructive signals, including different concentrations of RA and dietary amino acids, which allows cell adaptation to occur in a spatially defined manner. Analogous mechanisms guide the differentiation of epithelial cells as they migrate along the crypt–villus axis (45, 60), suggesting that it may be a general principle of cell adaptation to the SI; future studies could examine whether other immune cell types also mature along the crypt–villus trajectory.

Our transcriptional analysis of SI eosinophils at early and late stages of adaptation revealed many differentially expressed genes from multiple biological pathways. The enrichment of genes related to translation and glycolysis in the younger eosinophils (α4β7+) is consistent with the notion that tissue adaptation is an anabolic process, presumably involving the synthesis of proteins that support the specialized functions in the tissue. The upregulation of cholesterol biosynthetic genes at later stages is surprising; the role of cholesterol in eosinophil biology is largely unexplored and could have important implications for their survival and/or function in this tissue environment. The downregulation of genes involved in inflammation might indicate that eosinophils are polarizing toward a distinct regulatory state under homeostatic conditions, analogous to a subpopulation of regulatory eosinophils recently described in the lung (61). Since our analyses were based on RNA sequencing of bulk populations, they do not fully capture the phenotypic diversity of cells at these different stages. Future analyses based on single-cell sequencing, while exceptionally challenging for eosinophils due to their abundant RNases, would help address this issue (40).

One of the most distinctive features of the SI environment is its direct exposure to dietary nutrients. We therefore hypothesized that dietary nutrients, or signals derived from them, play a role in shaping eosinophil adaptation to this tissue. Consistent with this hypothesis, we found that RA, a metabolite of vitamin A, promoted the accumulation of villus-resident eosinophils via activation of RARs. This result is consistent with a recent study which proposed that specific microbes can limit intestinal eosinophil accumulation by suppressing RA production (34). It also reinforces the general importance of RA for guiding adaptation of cells to the SI, previously reported for epithelial cells, dendritic cells, and innate lymphoid cells, among others (62–64). Despite the importance of IL-5 for maintaining circulating eosinophils, loss of IL-5 only had a modest effect on eosinophil turnover in the SI, suggesting that eosinophils “switch” their dependence from IL-5 to RA after entry into this tissue.

While we observed that RA can directly promote survival of isolated SI eosinophils, our results also raise the possibility that RA can promote a developmental transition associated with villus residency, in line with its general role in cell differentiation (65). RA in the SI can be synthesized from vitamin A by enterocytes and dendritic cells (64), and future studies could dissect the contributions of these sources to eosinophil adaptation through cell type-specific ablation of RA synthesis genes. Whether the eosinophil dependence on RA is unique to the SI is unclear; it is tempting to speculate that each tissue type produces a distinct combination of signals to guide eosinophil adaptation in a manner that is appropriate for function in that tissue.

In addition to regulation by RA, we unexpectedly found that elevated dietary amino acids in a high-protein diet accelerate eosinophil turnover and specifically suppress accumulation of villus-resident eosinophils. Adoptive transfer experiments suggested that this effect is not attributable to altered eosinophil development in the bone marrow and most likely reflects altered signaling within the SI. While we observed increased eosinophil apoptosis on high-protein diet, indicating that survival is reduced, it remains possible that changes to eosinophil differentiation or migration also contribute to reduced accumulation of CD22+ eosinophils in the villi. The exact mechanism(s) by which amino acids influence eosinophil adaptation to the SI will require further investigation; though amino acids have diverse direct effects on immune cells (66), our data suggest that the effect is indirect and not dependent on RARs, AHR, or microbiota (*SI Appendix*, Fig. S4). Future studies should test the involvement of amino acid sensors, such as CaSR and GPRC6A, as well as signaling molecules that are regulated by dietary amino acid abundance (67).

The distinct effects on villus-resident and crypt-resident SI eosinophils after RAR inhibition or high-protein diet raise the possibility that eosinophil adaptation is tied to enterocyte programs that exhibit graded expression along the crypt–villus axis, referred to as enterocyte zonation (45, 46). For example, antimicrobial genes are enriched among enterocytes near the bottom of the villus, while genes involved in amino acid, carbohydrate, and lipid absorption are sequentially enriched from the center to the top of the villus (45, 46). These zonation patterns arise as enterocytes gradually migrate and mature under the control of opposing Wnt and BMP gradients along the crypt–villus axis (60). The higher expression of RA synthesis genes *Aldh1a1* and *Rdh7* in mature enterocytes, which are located in the upper portion of the villus, is consistent with eosinophils encountering higher levels of RA as they move into this region (68). Similarly, it is possible that enterocyte-derived signals that are expressed in conjunction with amino acid handling genes near the center of the villus are involved in eosinophil regulation in response to dietary amino acid abundance. Thus, the spatial pattern of eosinophil adaptation to the SI might be intimately linked to the parallel pattern of enterocyte zonation.

Our findings in eosinophil-deficient ΔdblGATA mice support a role for SI eosinophils in tissue architecture and local immunoregulation. The observation of decreased villus area in ΔdblGATA mice is consistent with similar findings in a recent report (33). It might be a consequence of direct action of eosinophil products, such as matrix metalloproteinases, on the extracellular matrix, or it might be indirectly mediated via eosinophil cross talk with other cells, such as fibroblasts (69). This structural alteration might partially contribute to the reduced numbers of monocytes, dendritic cells, T cells, and other immune cells in the LP of ΔdblGATA mice. In addition, SI eosinophils might directly or indirectly support recruitment or maintenance of these immune cells via production of chemokines and other signals. We suspect that the villus-resident eosinophils are primarily responsible for these functions due to their greater degree of tissue adaptation, but definitively testing this hypothesis requires the development of new tools to selectively ablate specific eosinophil subsets.

Unexpectedly, we were not able to reproduce some of the findings reported by other researchers who studied ΔdblGATA or PHIL (eosinophil-deficient) mice, including observations of altered epithelial migration, accelerated gut motility, and increased intestinal permeability (33, 34). Since some of these functions were reported to depend on microbiota, we suspect that the discrepancy can be explained by differences in microbiota composition across mouse facilities. It will be important for future studies to continue to define the microbiota species that influence eosinophil functions, determine their prevalence in different mouse facilities, and ascertain the mechanisms by which they exert this influence.

Our observations of eosinophil regulation by nutrient-derived factors add to a growing body of evidence linking eosinophils to different aspects of nutrition and metabolism. Earlier studies had demonstrated effects of fasting or high fat diet feeding on eosinophil homeostasis, as well as eosinophil roles in lipid and glucose metabolism (18, 21, 33, 41, 50). The significance of these surprising associations is uncertain; from an evolutionary perspective, they might reflect a fundamental, conserved role of eosinophils in immune-metabolic cross talk. For example, remodeling of eosinophil populations in response to shifts in diet composition might help steer local immune responses in a direction that is more suitable for a particular metabolic state. It is also possible that a dietary shift could be interpreted by the host as a sign of a parasite infection which has evaded direct detection; a subsequent remodeling of the local eosinophil populations might help optimize defenses against this threat.

In all, our study demonstrates how eosinophil adaptation to the SI under homeostatic conditions is regulated by different nutrient-derived signals and further highlights eosinophil contributions to tissue structure and immunoregulation in this tissue. Whether analogous signaling mechanisms orchestrate eosinophil adaptation in other tissues and whether these are conserved from mouse to human are important questions for future investigation. Diet-dependent regulation of eosinophils could have important implications either for normal physiology or disease states associated with eosinophil accumulation, such as eosinophilic gastroenteritis. Thus, our study raises several key questions at the intersection of eosinophil biology, tissue homeostasis, and nutrition.

## Materials and Methods

Additional methods are provided in *SI Appendix*.

### Animals and Diets.

All animal experiments were performed in accordance with institutional regulations after protocol review and approval by Yale University’s Institutional Animal Care and Use Committee. The following strains were originally obtained from Jackson Laboratories and subsequently bred in house: C57BL/6J (stock no. 000664), B6 CD45.1 (stock no. 002014), BALB/cJ (stock no. 000651), ΔdblGATA (stock no. 005653), and Red5 (R5/R5) (stock no. 030926). *Il1rl1*^−/−^ mice were provided by Andrew McKenzie (MRC Laboratory of Molecular Biology). Germfree C57BL/6 mice were bred and maintained in sterile conditions in Class Biologically Clean isolators (Class Biologically Clean, Madison, WI, USA). Dietary interventions in germfree mice were performed in microisolator cages (Isocage 72P; Techniplast). Animals were exposed to a 12:12 hour light/dark cycle (light from 7 AM to 7 PM). Male and female mice of 6 to 12 wk of age were used for all experiments. Age- and sex-matched mice were used for all experiments, and cohoused littermates were used whenever possible, including WT control littermates for experiments involving genetically modified strains.

### Diet Experiments.

Mice had free access to food except during fasting and food restriction experiments. For overnight fasting experiments, food was removed in the afternoon, and mice were analyzed the following morning. For every other day fasting experiments, mice were subjected to repeated cycles of 24 h of fasting followed by 24 h of ad lib feeding for a total duration of 15 d. For food restriction experiments, mice were provided 60% of the amount of food consumed by control mice fed ad libitum for a total duration of 15 d. Removal or provision of food was done in the afternoon.

Regular mouse diet was Teklad Global 18% Protein Rodent Diet (2018S) from Harlan Laboratories. Custom diets with variable macronutrient composition (high carbohydrate, high protein, and high fat) were purchased from Envigo with irradiation and vacuum packing. Complete compositions of all purchased custom diets are shown in *SI Appendix*, Table S1. Custom diets with and without Indole-3-carbinol (I3C) were purchased from Research Diets, Inc. (*SI Appendix*, Table S1). Custom diets supplemented with variable amounts of casein or purified amino acids were prepared in-house in powder form, starting from a concentrated 30% basal mixture (Envigo, *SI Appendix*, Table S1) that was supplemented with variable amounts of corn starch (Earthborn Elements), casein (Envigo), or purified individual amino acids (Purebulk Inc. or Hard Eight Nutrition LLC) to make the complete diets with the indicated protein and/or amino acid compositions. Individual amino acids were mixed together to match the natural composition of casein (70). Powdered diets were provided in plastic containers covered by metal feeder shields (Unifab Corp) to limit spillage. Mice were fed custom diets for 15 d unless indicated otherwise.

### BrdU Labeling In Vivo.

BrdU labeling was initiated by intraperitoneal (I.P.) injection of 2 or 3 mg of BrdU. In BrdU pulse–chase experiments, mice were provided BrdU in drinking water (0.8 mg/mL) for a 24 h period, then switched to plain water for the indicated number of days. In continuous labeling experiments, mice were maintained on BrdU-containing water for the indicated number of hours or days. BrdU water was replaced every 3 d and protected from light.

### BMS493 Treatment.

Mice received daily I.P. injections of BMS493 (MedChemExpress, 220 µg in 25 µL of DMSO) for 8 or 12 d. Controls received DMSO alone.

### Antibiotic Treatment.

Mice received vancomycin (500 mg/L) (Cayman Chemical), ampicillin (1 g/L) (Sigma Aldrich), neomycin (1 g/L) (Sigma Aldrich), and metronidazole (1 g/L) (Sigma Aldrich) in drinking water starting at 6 wk of age for a total of 4 wk. Antibiotic water was replaced weekly and protected from light. Mice were fed control or high-protein diets for the final 15 d of antibiotic treatment.

### Gut Transit Time.

To measure gut transit time, 18 mg of carmine red dye in 0.3 mL of 0.5% methylcellulose was administered by oral gavage to singly housed mice. The time until the appearance of the first red fecal pellet (corresponding to the gut transit time) was recorded.

### Intestinal Permeability (FITC-Dextran Assay).

To measure intestinal permeability, mice were fasted for 3 h and then received 5 to 10 mg of 4 kDa FITC-dextran (Sigma) in 0.2 mL phosphate-buffered saline (PBS) by oral gavage. One hour later, plasma was collected, and FITC fluorescence (excitation at 488 nm, emission at 538) in the plasma was measured with a BioTek microplate reader. Background fluorescence signal was determined in plasma samples from untreated control mice and subtracted from the experimental values.

### IL-5 Delivery and Eosinophil Adoptive Transfer.

To boost eosinophil production in donor B6 CD45.1 mice, IL-5 overexpression was induced by hydrodynamic delivery of pCAG-IL-5 plasmid (generously provided by Jun-ichi Miyazaki) (59). The plasmid was injected intravenously (I.V., via the retro-orbital sinus) at 2.5 mg/kg in 0.1 mL/g of normal saline, in 6 to 9 s. Two weeks later, mice were killed, and splenic eosinophils were isolated under sterile conditions. To leave eosinophils “untouched,” eosinophils were isolated by negative selection using anti-biotin MicroBeads and LS columns (Miltenyi Biotec) according to the manufacturer’s protocol. Cells were incubated with the biotinylated antibodies against the following antigens: NK1.1, CD90.2, CD8a, B220, MHCii (IA/IE), Ly6g (clone 1A8), and Ter119 (see *SI Appendix*, Table S2 for antibody details). Flow cytometric analysis confirmed that eosinophil purity after isolation was >90%. Approximately 25 × 10^6^ eosinophils were adoptively transferred by intravenous injection into B6 CD45.2 recipients (hosts) which had been fed either control or high-protein diets for the preceding 2 wk. Eight days after transfer, mice were killed, and the proximal SI LP was isolated for analysis of donor (CD45.1+) and host (CD45.2+) eosinophils by flow cytometry, as described earlier.

### Ex Vivo Culture of Isolated LP Cells with Different Concentrations of Amino Acids.

Isolated LP cells from the proximal SI were further purified by Percoll density gradient centrifugation. Cells were resuspended in 40% isotonic Percoll (GE Healthcare, diluted with PBS) and carefully laid on top of 75% isotonic Percoll. After centrifugation for 20 min at 800 g (without brake), cells were collected from the interface between the 40% and 75% Percoll layers. The cells were washed twice with cold Roswell Park Memorial Institute (RPMI) media and then cultured at a density of 4 × 10^5^ cells in 125 μL of modified complete RPMI media containing 10% FBS and a variable amount of amino acids for 48 h at 37 °C with 5% CO_2_. The RPMI media were prepared with either 1, 2, 4, 6, 8, or 12 times the normal amount of amino acids provided in standard Minimum Essential Medium (MEM) media (Gibco). Live cells (exhibiting weak staining with Zombie Yellow dye) were quantified by flow cytometry before and after culture, and cell viability after 48 h was calculated as a percentage of the initial input.

### Ex Vivo Culture of Isolated LP Eosinophils with RA.

Eosinophils were isolated from proximal SI LP by Percoll (described above) and by negative selection with anti-biotin MicroBeads and MS columns (Miltenyi Biotec) according to the manufacturer’s protocol. Cells were incubated with Fc block and biotinylated antibodies against the following antigens: IgA, MHC class II, Ly6g, CD115, B220, CD140a, CD90.2, and CD8a. Eosinophils were enriched to >90% purity. Approximately 50 × 10^4^ eosinophils per well were cultured in complete RPMI with 100 nM all-*trans*-RA (Sigma) or DMSO (vehicle control) alone. Eosinophil viability was measured by flow cytometry on day 3 of culture (% of eosinophils exhibiting weak staining with Zombie Yellow dye).

### RNA Sequencing.

Sequencing libraries were constructed with SMARTer® Stranded Total RNA-Seq Kit v3–Pico Input Mammalian (Takara Bio) according to the manufacturer’s instructions. Three nanograms of total RNA were used as input. Library size was determined by Agilent High Sensitivity RNA ScreenTape (Agilent Technologies). Library concentration was determined by the KAPA Library Quantification Kit (Kapa Biosystems) according to the manufacturer’s instructions. Paired-end sequencing was performed with NextSeq 1000/2000 P2 Reagents (100 Cycles) v3 on a NextSeq 2000.

### RNA Sequencing Analysis.

Trimmed and processed sequencing reads were pseudoaligned to the mouse mm10 genome build using Kallisto (71). Differential expression, defined as a fold change ≥ 1.5 and an adjusted *P*-value ≤ 0.05, was determined using DESeq2 (72). Genes with extremely low expression (average normalized count of 10 or less) were excluded from the analysis. K-means clustering analysis was performed with Morpheus (https://software.broadinstitute.org/morpheus). Heatmaps were generated in R (73). Gene ontology enrichment analysis was performed using DAVID Bioinformatics Resources 6.8 (74). The Volcano plot was made using the fold changes and adjusted *P*-values from DESeq2 (75).

### Statistical Analyses.

For most experiments, statistical significance was determined by two-sided Student’s *t* test. Significance in Fig. 1*D* was determined by linear regression analysis. Significance in RNA-seq was determined by differential expression analyses as described above.

## Supplementary Material

Appendix 01 (PDF)

## Data Availability

The RNA-seq data have been deposited at GEO (https://www.ncbi.nlm.nih.gov/geo/) under accession number GSE236132 (76).
