# Supplementary material for: Nutrient-derived signals regulate eosinophil adaptation to the small intestine
Source: Proc Natl Acad Sci U S A. 2024 Jan 25;121(5):e2316446121. doi: 10.1073/pnas.2316446121 (PMC10835075; doi:10.1073/pnas.2316446121)
Supplement: Supplementary file 1 — Appendix 01 (PDF) [file pnas.2316446121.sapp.pdf]

Supporting Information for

Nutrient-derived signals regulate eosinophil adaptation to the small intestine

Vassily I. Kutyavin, Lisa L. Korn, Ruslan Medzhitov

Ruslan Medzhitov
Email: Ruslan.medzhitov@yale.edu

This PDF file includes:

Supporting Text
Figures S1 to S5
Tables S1 to S3
SI References

Supporting Information Text

Additional Methods

Epithelial cell & lamina propria isolation (for qPCR and flow cytometry)

Single cell suspensions of epithelial and lamina propria cells from the small intestine were prepared as described previously (3). For most experiments, only the proximal half of the small intestine tissue was collected. Unless otherwise indicated, isolation was performed in the morning. For separate isolation of duodenum, jejunum, and ileum, the most proximal 4 cm of the tissue was collected as duodenum, the proximal half of the remaining tissue was collected as jejunum, and the distal half was collected as ileum. Briefly, after removal of Peyer's patches, the intestine tissue was opened longitudinally, washed extensively in phosphate-buffered saline, cut into 1-2 cm pieces and incubated in RPMI media containing 5 mM EDTA, 0.145 mg/mL DTT, and 3% FBS at 37°C with 5% CO_2 for 20 minutes with agitation. The tissue pieces were washed three times in serum-free RPMI media containing 2 mM EDTA by vigorous shaking. All of the resulting media (containing the epithelial fraction) was collected and pooled. A fraction of the collected epithelial cells was taken for RNA isolation. The remaining tissue (containing lamina propria) was minced and incubated in RPMI containing 0.1 mg/mL Liberase TL and 0.05% DNase at 37°C with 5% CO_2 for 30 minutes with agitation. The resulting suspension was then passed through 70 and 40 µm strainers to isolate lamina propria cells for flow cytometry.

Flow cytometry

Single cell suspensions were first stained with Zombie Yellow viability dye (Biolegend) according to the manufacturer's instructions. Next, cells were incubated with anti-CD16/32 (Fc block) and fluorochrome-conjugated antibodies directed at cell surface antigens for 20 minutes at 4° C (see Table S2 for full list of antibodies). BrdU staining was performed with a FITC BrdU Flow Kit (BD Biosciences) according to the manufacturer's instructions. Staining for transcription factors was performed using the Foxp3 / Transcription Factor Staining Buffer set (Ebioscience). 123count eBeads (Invitrogen) counting beads were added to the cell suspensions to facilitate absolute cell number quantification. Flow cytometric analysis was performed on a BD Biosciences LSR II or FACSymphony cell analyzer with FACSDiva software. Downstream data processing and analysis was done in FlowJo (FlowJo, LLC).

Immune cell populations were identified as singlet, live, CD45+ cells. Eosinophils were additionally CD11b+ SiglecF+ MHC class II (MHCii)- Ly6g-. Neutrophils were CD11b+ Ly6g+ SiglecF-. ILCs were lineage negative (in Figure S2: CD11b, CD11c, CD19, Gr-1, NK1.1, Ter119; in Figure S4: CD3, CD5, CD8a, CD8b, CD11b, CD11c, CD19, Gr-1, TCRβ, Ter119) and Gata3+ or Rorγt+ (ILC2 or ILC3, respectively). αβ T cells were TCRβ+ and CD4+ Foxp3+ (Tregs), CD4+ RORγt+ (Th17), CD4+ Gata3+ (Th2), or CD8α+ (CD8 T cells). γδ T cells were γδTCR+. Plasma cells were IgA+ and negative for CD11c, CD11b, and B220. Ly6c+ monocytes were CD11b+ CD64+ SiglecF- Ly6G- MHCii- Ly6c+. Macrophages were CD11b+ CD64+ SiglecF- Ly6G- MHCii+ Ly6c-. Dendritic cells were CD11c+ MHCii+ CD103+ and further defined as CD11b+ or CD11b-.

Cell sorting

Eosinophils were sorted from single cell suspensions of proximal small intestine lamina propria. Cells were stained with Zombie Yellow and surface makers as in flow cytometry. Following staining, DAPI was added (0.05 ug/ml) so that cells that died after the initial staining could be excluded. Sorting was performed on a BD Aria II using a 100 µM nozzle

and eosinophil subsets were sorted into 100% fetal bovine serum at 4°C. Each subset sample contained 225,000-415,000 sorted eosinophils from 2 pooled mice. After sorting, RNA was isolated immediately.

Isolation of immune cells from other tissues

For some organs, tissue eosinophil frequency (shown as "percent of non-circulating CD45+ cells") was determined by exclusion of circulating eosinophils. Briefly, mice were injected intravenously with 3 µg anti-CD45 antibody conjugated to Alexa Fluor 700 approximately 3 minutes prior to sacrifice to label blood-exposed cells, and these cells were identified by flow cytometry and excluded from the subsequent analysis.

Adipose tissue: Perigonadal adipose tissue was isolated and minced with scissors, then incubated in Ham's F-10 media containing 2 mM L-glutamine, 15 mg/mL bovine serum albumin (BSA), and 1 mg/mL Type 1 Collagenase (Worthington) for 30 minutes at 37°C, shaking. The resulting cell suspension was then briefly vortexed, passed through a 70 µm strainer, and washed twice with Ham's F-10 media prior to staining for flow cytometry.

Blood: Blood was collected by retro-orbital puncture into EDTA-coated tubes and lysed with Ammonium-Chloride-Potassium (ACK) buffer (6 minutes at room temperature), then washed with PBS prior to staining for flow cytometry.

Liver: After intravenous labeling with anti-CD45 (see above), the whole liver was isolated and minced with scissors, then incubated in Hank's Balanced Salt Solution (HBSS) containing 0.2 mg/mL BSA, 0.01 mg/mL DNase I, and 1 mg/mL Type IV Collagenase (Worthington) for 30 minutes at 37°C, shaking. The resulting cell suspension was then briefly vortexed, passed through a 70 µm strainer, and centrifuged. The pellet was then resuspended in 33% Percoll (diluted with PBS) and centrifuged for 20 minutes at 800 g, room temperature, without brake. The resulting pellet was then lysed with ACK buffer (6 minutes at room temperature), then washed with PBS twice before staining for flow cytometry.

Mesenteric lymph nodes: The entire chain of mesenteric lymph nodes was dissected from surrounding tissue and manually disrupted through a 70 µm strainer to create a single cell suspension.

Peritoneal cavity: To isolate peritoneal cells, the peritoneal cavity was exposed and injected with 4.5 mL of RPMI media containing 3% FBS using a 30 gauge needle. The peritoneal cavity was vigorously agitated for one minute to dislodge cells, then the fluid (containing peritoneal cells) was collected.

Stomach and colon: Single cell suspensions of stomach and colon lamina propria were isolated in the same manner as the small intestine.

Spleen: Spleens were manually disrupted through a 70 µm strainer, incubated with ACK lysis buffer for 5 minutes at room temperature, resuspended in fresh media, and filtered again as needed prior to downstream applications.

Thymus: Thymus was dissected, gently blotted briefly on a Kimwipe to remove excess blood or fat if present, shredded with tweezers to create a single cell suspension, and filtered through a 70 µm strainer prior to staining for flow cytometry.

Histology, immunofluorescence/immunohistochemistry, and TUNEL
For most procedures, freshly isolated small intestine was fixed in 10% formalin overnight at room temperature. The tissue was then embedded in paraffin, sectioned at 5 µm thickness, deparaffinized with xylene and rehydrated through graded concentrations of ethanol in water. Sections were then stained with hematoxylin and eosin (H&E), or processed for immunofluorescence/immunohistochemistry or TUNEL. For MBP staining, antigen retrieval was performed with Digest-All™ 3 pepsin for 30 minutes at 37° C, followed by blocking (2.5% normal goat serum and 2.5% normal donkey serum) for 1 hour at room temperature, and then incubation with rat anti-MBP antibody (clone MT2-14.7.3, 1 µg/mL, purchased from the laboratory of Elizabeth Jacobsen, Mayo Clinic) overnight at 4° C. This was followed by incubation with secondary goat anti-rat IgG conjugated to Alexa Fluor 568 (or similar) for 30 minutes at room temperature. Terminal deoxynucleotidyl transferase dUTP nick end labeling (TUNEL) was done with In Situ Cell Death Detection Kit (Roche) per the manufacturer's instructions. For pretreatment of the tissue sections, the permeabilization solution was used. DAPI staining was performed at 300 nM for 3 minutes. Samples were mounted with Fluoromount G (Southern Biotech).

BrdU immunohistochemistry to quantify epithelial migration was performed by Yale Pathology Tissue Services. Small intestine sections were deparaffinized and rehydrated as described above, then denatured by incubation with 1N HCl at 37° C for 30 minutes. Epitope retrieval was then performed by incubation with trypsin. Next, the sections were incubated with H_2O_2 to quench endogenous peroxidase activity. Then the sections were incubated with primary anti-BrdU antibody (Sigma #B2531), followed by incubation with secondary 'MACH 2' antibody reagent (Biocare Medical) conjugated to horseradish peroxidase. The sections were developed by applying 3,3′-Diaminobenzidine (DAB), followed by counterstaining with hematoxylin, dehydration, and mounting with resinous mounting media.

For CD22 and BrdU immunofluorescence staining in eosinophils, freshly isolated proximal small intestine was immediately frozen in optimal cutting temperature (OCT) compound (Sakura Finetek). The tissue was sectioned at 10 µm thickness, air dried for 30 minutes, and fixed with 10% formalin for 20 minutes. For CD22 staining, the sections were then blocked and permeabilized with Tris-buffered saline (TBS) containing 0.025% Triton X-100, 1% bovine serum albumin, 2.5% normal donkey serum, and 0.3 M glycine for 1 hour at room temperature. This was followed by incubation with goat anti-CD22 (R&D Systems, #AF2296, 0.67 µg/mL) and rat anti-MBP (0.5 µg/mL) antibodies in the same buffer as above (without glycine) overnight at 4° C. Secondary antibody incubation, DAPI staining, and sample mounting was performed as described earlier. For BrdU staining, fixed sections were incubated with 2N HCl for 30 minutes at 37° C, neutralized by two washes in 0.1 M borate buffer (pH 8.5), followed by permeabilization with 0.2% Triton X-100 in PBS for 30 minutes at room temperature. The samples were then blocked with 2.5% normal donkey serum and 2.5% normal goat serum for 1 hour at room temperature. This was followed by incubation with anti-BrdU antibody conjugated to APC (BD Pharmingen #51-23619, 1:200) and rat anti-MBP antibody (1 µg/mL) for 2 hours at room temperature. Secondary antibody incubation, DAPI staining, and sample mounting was performed as described earlier.

Imaging of CD22 staining (in combination with MBP and DAPI) was performed with a Leica Stellaris 8 confocal microscope, using 40X/1.3 or 63X/1.4 oil objectives. Imaging of other immunofluorescence staining was performed with a Leica DMI6000 B widefield microscope, using 10X, 20X, or 40X air objectives. LAS X software (Leica) was used for

image acquisition and export in tiff format. ImageJ/Fiji software (NIH) was used for image analysis.

Eosinophils were identified based on the presence of cytoplasmic MBP staining. Eosinophils were quantified per 'region', defined as an area of the tissue section that corresponds to 1 mm of longitudinal intestinal length. Apoptosis was indicated by overlap between TUNEL and DAPI staining. BrdU+ eosinophils were identified first by finding overlap of MBP and BrdU signal (at 20X on the widefield microscope). BrdU incorporation was then visually verified by detecting overlap between BrdU staining and nuclear DAPI staining in the eosinophil. Only eosinophils with BrdU signal above a certain threshold (equivalent to signal observed on day 3 after BrdU labeling) were counted in the analysis in order to exclude eosinophils marked by residual BrdU remaining in progenitors after termination of the BrdU pulse. Eosinophil position along the crypt-villus axis was determined by measuring its relative distance from the villus base (position '0') and villus tip (position '100'). Eosinophils located below the villus base (in the vicinity of the crypts) were identified as crypt eosinophils, while those located above were identified as villus eosinophils. BrdU incorporation analysis was performed in a blinded manner.

Imaging of H&E staining and BrdU immunohistochemistry for quantification of epithelial migration was done with an Olympus BX40 microscope, using 10X/0.25 or 20X/0.40 air objectives. Images were exported in JPG format. ImageJ software (NIH) was used to quantify villus length, area, and epithelial migration. Villus length was measured as the distance from the villus base to the villus tip. Villus cross-sectional area was measured between the villus tip and the base. Epithelial migration distance was measured as the distance from the villus base to the cluster of BrdU+ epithelial cells located closest to the villus tip. At least 30 villi were measured in each sample to obtain a mean value. All image analysis and quantification were done in a blinded manner.

RNA isolation and quantitative RT-PCR (qPCR)

Epithelial cell pellets or whole tissue pieces were homogenized in RNA-Bee or RNA STAT-60 reagents (both AMSBIO). Cell pellets were vortexed and tissue pieces were placed in Omni bead tubes and homogenized using a Bead Ruptor Elite bead mill homogenizer (Omni Intl). RNA was isolated with a Direct-Zol RNA MiniPrep Plus kit (Zymo Research) according to the manufacturer's instructions. RNA concentration was measured with a NanoDrop Eight Spectrophotometer. cDNA synthesis was performed with 1 μg of isolated RNA, 0.5 μg of oligo(dT)20 primer, 1 mM dNTPs, 10 mM DTT, and 50 units of RT SMART MMLV Reverse Transcriptase in First-Strand Buffer (Takara Bio). qPCR was performed with PerfeCTa SYBR Green (Quanta Bio) using the BioRad CFX384 platform. qPCR primer sequences used in this study are listed in Table S3.

The threshold value (CT) was used to calculate the relative abundance of mRNA. The mRNA abundance in each sample was expressed relative to the abundance of *Rpl13* (the reference gene).

To isolate RNA from sorted eosinophils, cells were lysed in 0.5 ml of Tri Reagent (Sigma-Aldrich), incubated at room temperature for 5 minutes, followed by addition of 100 μl of chloroform. The mixture was vigorously shaken by hand, incubated at room temperature for 8 minutes, then centrifuged at 12,000 g for 15 minutes at 4° C. The aqueous phase was isolated and mixed with an equal volume of ethanol. This mixture was added to a Zymo-Spin™ IC Column and further RNA isolation was conducted with the Direct-zol RNA Microprep Kit (Zymo Research) according to the manufacturer's instructions. RNA amount

and quality was measured with the High Sensitivity RNA ScreenTape assay (Agilent) according to the manufacturer’s instructions. The RNA integrity number (RIN) of the samples used for sequencing ranged from 6.3 to 7.6.

Supplemental Figures and Legends

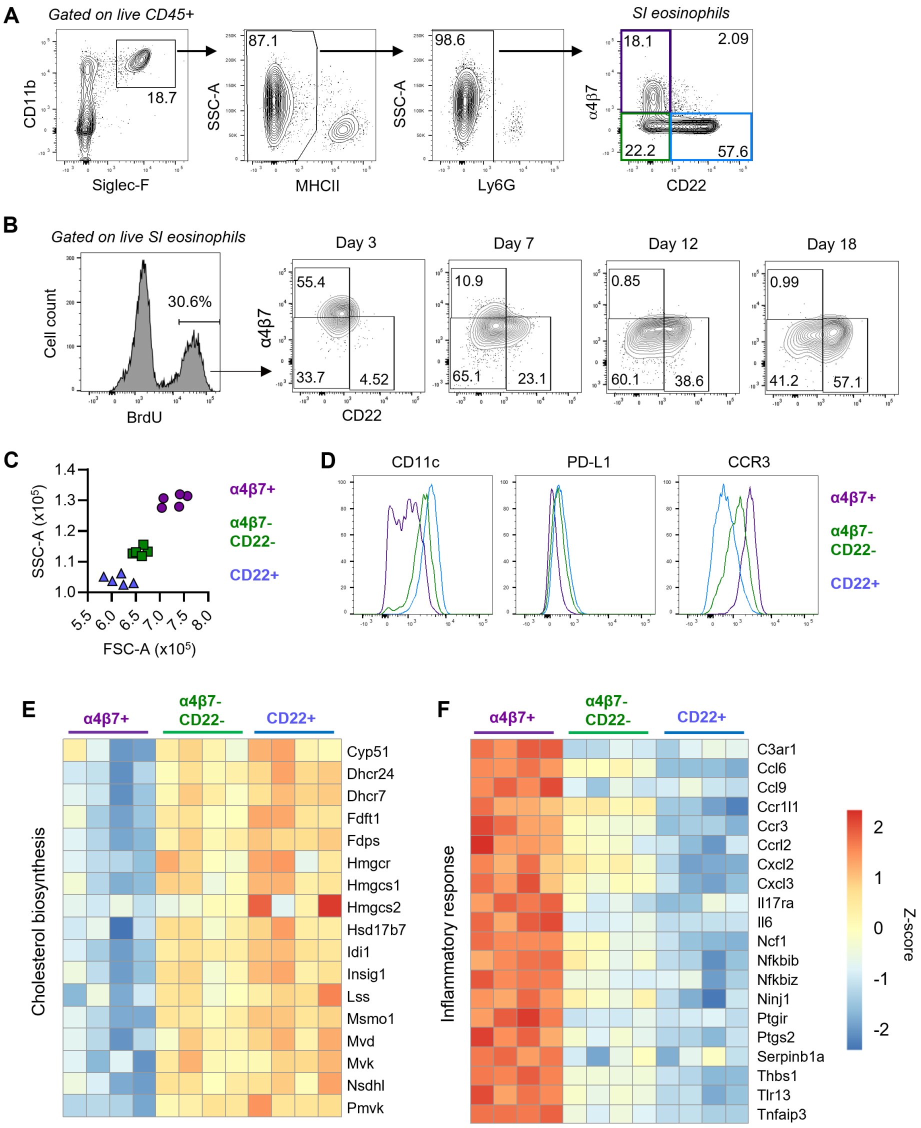

Figure S1. Eosinophil adaptation to the small intestine involves changes to morphology and gene expression.

(A) Flow cytometry gating to identify α4β7+, α4β7- CD22-, and CD22+ eosinophil subsets. Eosinophils (Zombie Yellow- CD45+ CD11b+ SiglecF+ MHCii- Ly6G-) were isolated from proximal SI lamina propria (LP).

(B) Flow cytometry gating to identify BrdU+ eosinophils (example shown is from day 7) and representative flow plots showing α4β7 and CD22 analysis on BrdU+ eosinophils from days 3, 7, 12, and 18 of BrdU pulse-chase protocol outlined in Fig. 1A.

(C) Forward scatter area (FSC-A, indicates cell size) and side scatter area (SSC-A, indicates internal complexity/granularity) of α4β7+, α4β7- CD22-, and CD22+ eosinophil subsets based on flow cytometric analysis (representative of 3+ experiments with 4-5 mice per group).
(D) Flow cytometry analysis of CD11c, PD-L1, and CCR3 staining on α4β7+, α4β7- CD22-, and CD22+ eosinophil subsets (representative of 3 experiments with 3-4 mice per group).
(E-F) Heatmaps of differentially expressed genes (fold change >1.5, adjusted p-value <0.05) in α4β7+, α4β7- CD22-, and CD22+ eosinophil subsets, highlighting examples from specific pathways (cholesterol biosynthesis and inflammatory response). The relative expression level of each gene, represented by Z-score (from -2 to +2), is indicated by color as shown in the legend.

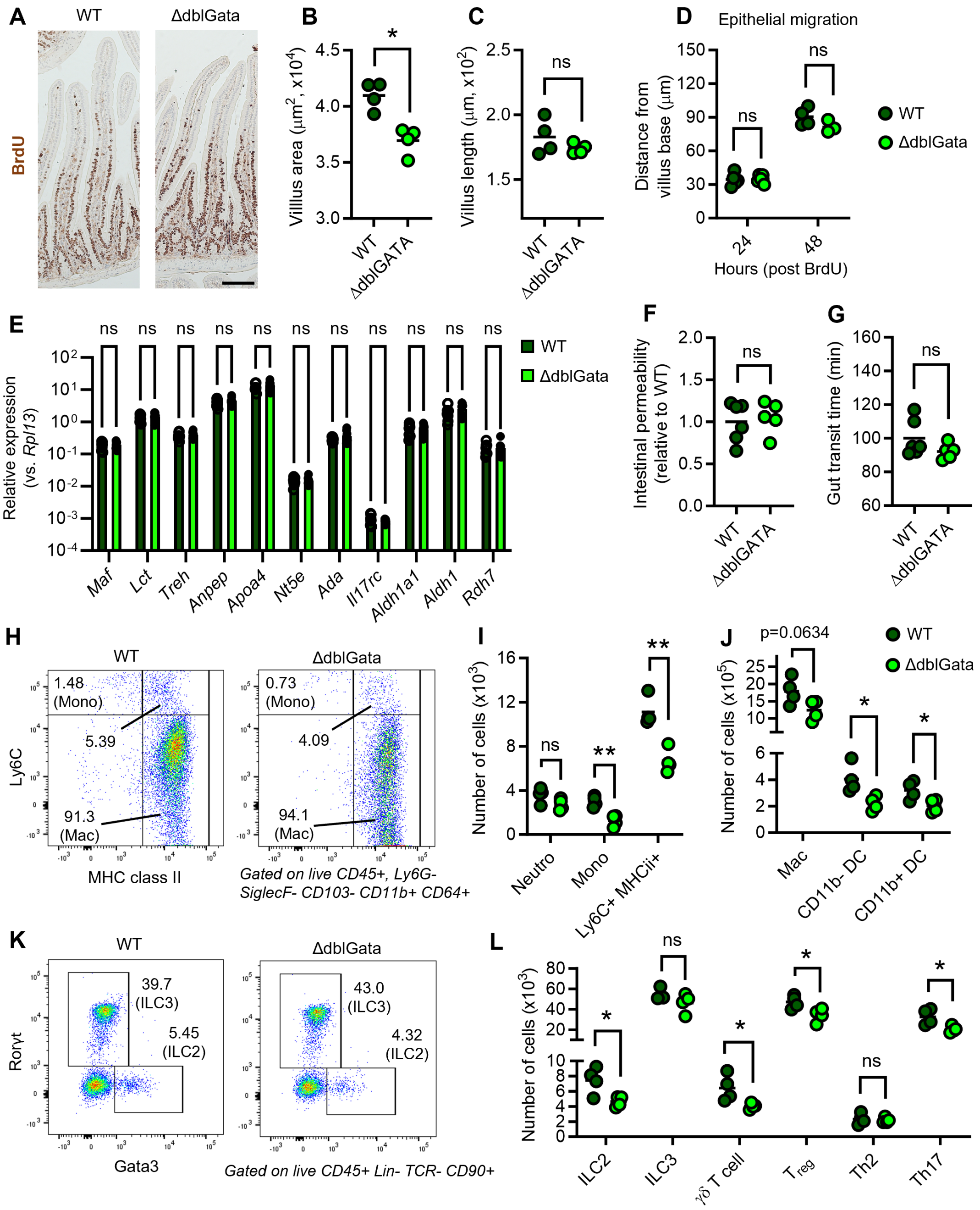

Figure S2. Eosinophil-deficient ΔdblGata mice exhibit decreased villus area and reductions of other immune cell populations in small intestine lamina propria.
(A-D) Representative microscopy images of proximal SI sections from WT and ΔdblGata mice, stained for BrdU (brown) and hematoxylin (light blue), after 48 hours of BrdU labeling (A; scale bar =, 100 μm). Quantification of villus cross-sectional area (B), villus length (C), and epithelial migration based on BrdU labeling (D) correspond to the same experiment. Representative of 2 experiments with 3-5 mice per group.
(E) Expression of *Maf* and c-Maf-dependent genes, relative to the reference gene *Rpl13*, in proximal SI epithelial fraction of WT or ΔdblGata mice, pooled from two experiments with 4-5 mice per group.

(F) Quantification of intestinal permeability in WT and ΔdblGata mice, indicated by FITC-dextran assay. Values are normalized to the mean value in the WT group. Representative of 3 experiments with 3-5 mice per group.
(G) Gut transit time in WT and ΔdblGata mice, measured by monitoring the passage of carmine red dye after oral administration. Representative of 3 experiments with 3-5 mice per group.
(H) Representative flow cytometry analysis of Ly6C and MHC class II (MHCii) expression on live CD45+ Ly6G- SiglecF- CD103- CD11b+ CD64+ cells in proximal SI LP of WT and ΔdblGata mice. Representative of 3 experiments with 3-5 mice per group.
(I-J) Quantification of neutrophils (Neutro), monocytes (Mono), macrophages (Mac), Ly6C+ MHCii+ cells, CD11b- and CD11b+ dendritic cells (DC) in proximal SI LP of WT and ΔdblGata mice. Representative of 3 experiments with 3-5 mice per group.
(K) Representative flow cytometry analysis of Rorγt and Gata3 expression among live CD45+ Lineage- TCR- CD90+ cells (innate lymphoid cells, ILCs) in proximal SI LP of WT and ΔdblGata mice. Representative of 2 experiments with 3-5 mice per group.
(L) Quantification of ILC2s, ILC3s, γδ T cells, Tregs, Th2s, and Th17s in proximal SI LP of WT and ΔdblGata mice. See methods for full gating strategy. Representative of 2 experiments with 3-5 mice per group.
All data were analyzed by Student's t-test. $**p < 0.01$, $*p < 0.05$, ns = not significant ($p > 0.05$).

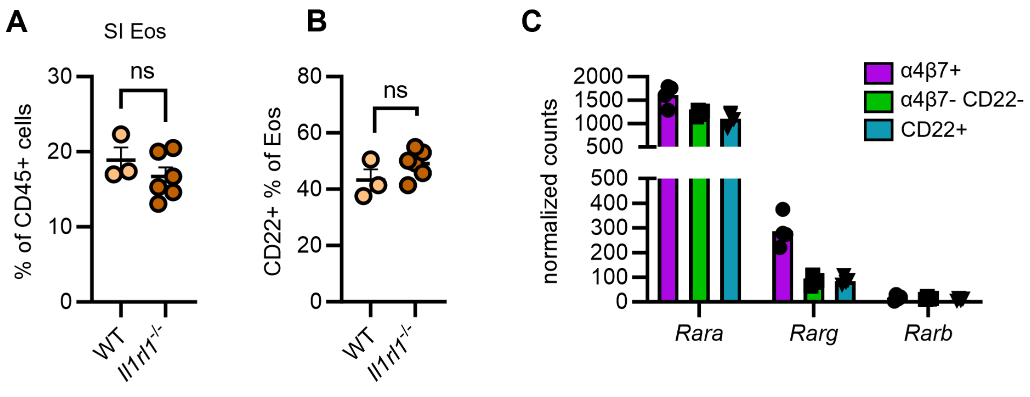

Figure S3. IL-33 signaling is not required for accumulation of CD22+ villus-resident eosinophils, and expression of RARs in SI eosinophil subsets.

(A-B) Eosinophil frequency (A, % of total live CD45+ cells) and percent of eosinophils that were CD22+ (B, by flow cytometry) in proximal SI lamina propria (LP) of WT and *Il1rl1*$^{-/-}$ (*St2*$^{-/-}$) mice. Representative of 3 experiments with 3-6 mice per group.

(C) Expression level (as normalized counts obtained from DESeq2) of *Rara*, *Rarb*, and *Rarg* in sorted α4β7+, α4β7- CD22-, and CD22+ eosinophil subsets.

Data in A-B are presented as mean ± SEM and were analyzed by Student's t-test. ns = not significant ($p > 0.05$).

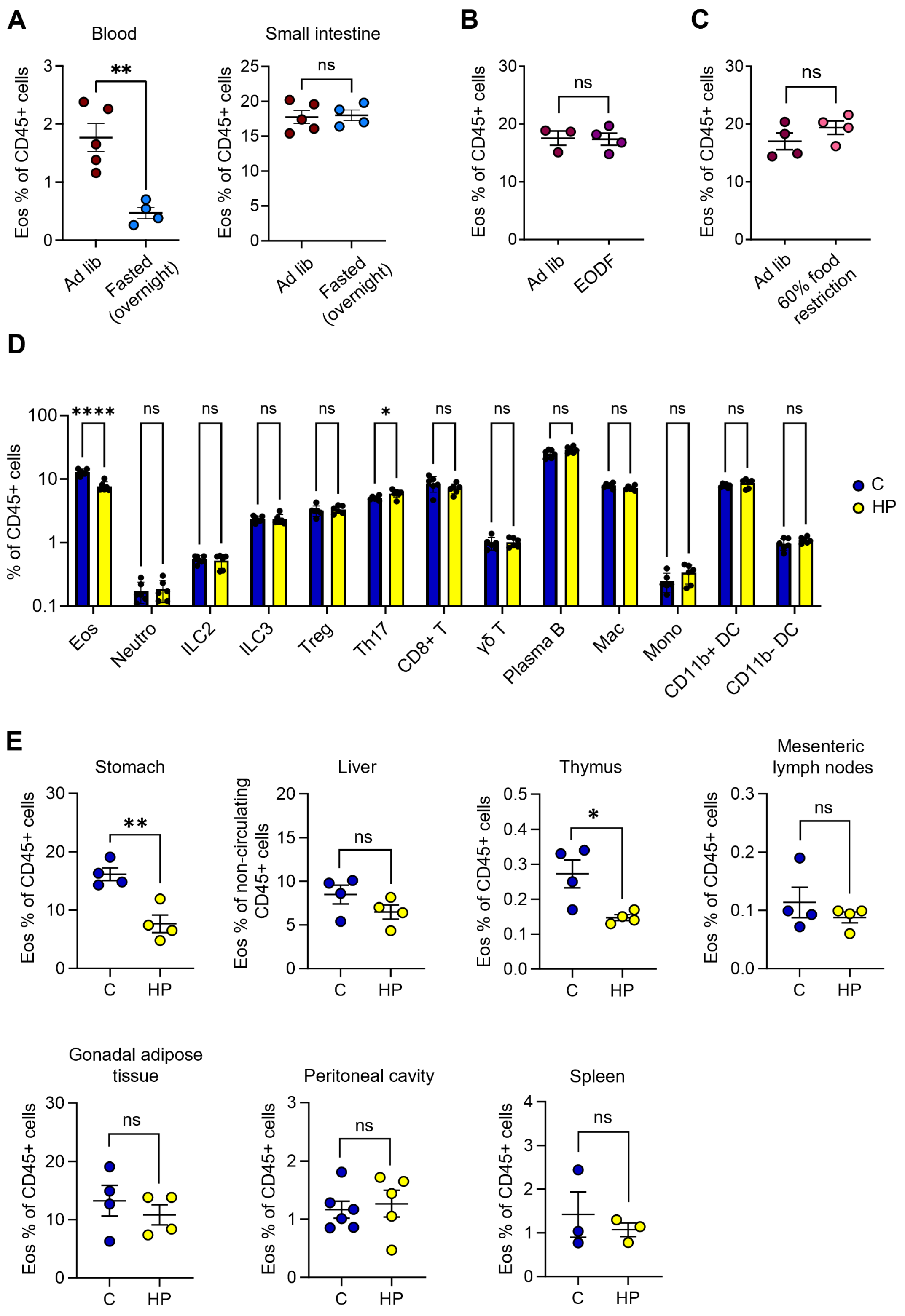

Figure S4. Food restriction does not reduce SI eosinophils, and the effect of high protein diet is limited to eosinophils in SI, stomach, and thymus.

(A) Eosinophil frequency in proximal SI lamina propria (LP) and blood of mice that were subjected to an overnight fast or allowed to eat ad libitum (Ad lib), representative of 3 experiments with 3 mice per group.
(B) Eosinophil frequency in proximal SI LP of mice subjected to every other day fasting (EODF) for 15 days or allowed to eat Ad lib, representative of 2 experiments with 4 mice per group.
(C) Eosinophil frequency in proximal SI LP of mice subjected to food restriction (60% of normal intake) or allowed to eat Ad lib for 15 days, representative of two experiments with 3-4 mice per group.
(D) Frequencies of eosinophils (Eos) and other immune cells in proximal SI of mice fed control ("C") or high protein ("HP") diet for 15 days, representative of 3 experiments with 4-5 mice per group (Neutro = neutrophil, Mac = macrophage, Mono = monocyte, DC = dendritic cell)
(E) Eosinophil frequencies in the indicated tissues in mice fed C or HP diets for 15 days. Each plot is representative of at least 2 experiments with 3-4 mice per group.
Data are presented as mean ± SEM. All data were analyzed by Student's t-test. $**p < 0.01$, $*p < 0.05$, ns = not significant ($p > 0.05$).

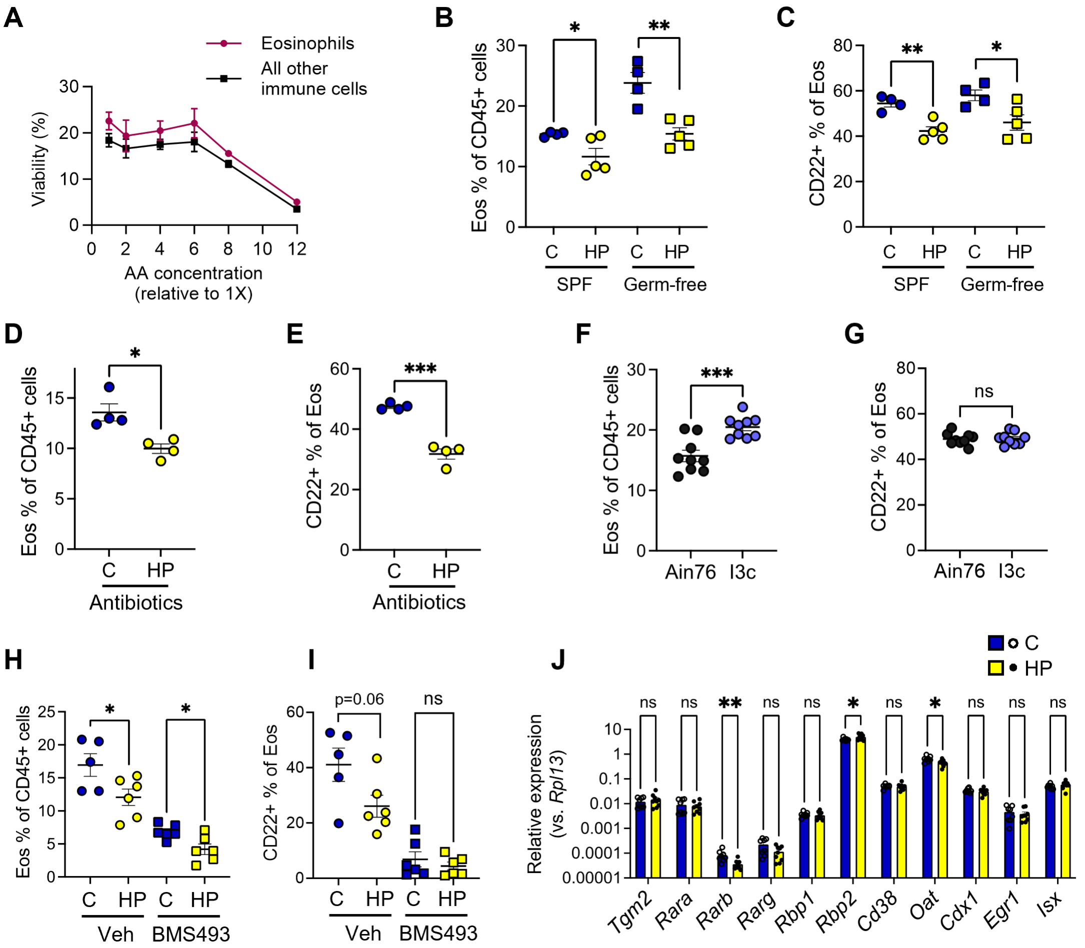

Figure S5. The effect of high protein diet on SI eosinophils is likely to be indirect and independent of several known regulators of SI eosinophil adaptation.

(A) SI lamina propria (LP) cells were cultured *ex vivo* with the indicated concentrations of amino acids (1X is equivalent to the standard concentration of amino acids in MEM media). Graph shows the viability of eosinophils compared to the average of all other immune cells after 48 hours of culture, representative of 2 experiments.

(B-C) SI LP eosinophil frequencies and percent of eosinophils that were CD22+ in germ-free and SPF mice after 15 days of feeding with control (“C”) or high protein (“HP”) diet, representative of 3 experiments with 4-6 mice/group.

(D-E) SI LP eosinophil frequencies and percent of eosinophils that were CD22+ in mice treated with a cocktail of broad spectrum antibiotics for two weeks prior to and during feeding with the indicated diets (15 days), representative of 3 experiments with 4 mice per group.

(F-G) SI LP eosinophil frequencies and percent of eosinophils that were CD22+ in mice fed a diet containing the AHR ligand I3C or a control diet (AIN76) for 15 days, data pooled from 2 experiments with 4-5 mice/group.

(H-I) SI LP eosinophil frequencies and percent of eosinophils that were CD22+ in mice treated (daily) with either BMS493 or vehicle control during the last 12 of 15 days of feeding with the indicated diets, data pooled from 2 experiments with 2-3 mice/group.

(J) Relative expression of retinoic acid receptor target genes (relative to *Rpl13*) in SI epithelial fraction of mice fed the indicated diets (15 days).

Data are presented as mean ± SEM. All data were analyzed by Student's t-test. ∗∗∗$p < 0.001$, ∗∗$p < 0.01$, ∗$p < 0.05$, ns = not significant ($p > 0.05$).

Supplemental Tables

Table S1. Nutritional composition of custom diets used in this study

Table S1. Nutritional composition of custom diets used in this study.

Ingredients (g/kg)	High carb/low protein (8%) diet (TD.170403)	High carb/normal protein (18%) diet (TD.170404)	High protein (70%)/low carb diet (TD.170405)	High fat/low carb diet (TD.220264)	30% Basal Mix (TD.210031)	Modified AIN-76A diet with I3C	Modified AIN-76A diet without I3C (control)
Casein (~87% protein content)	91.5	200	800	309	305	239.1	239.1
L-Cystine				4.5			
DL-methionine						3.42	3.42
Corn Starch	317	260	6.398		8.326	446.6	446.6
Maltodextrin	132	132	12	22	40	129.5	129.5
Sucrose	270.498	220.898	5	18	16.667	0	0
Vegetable shortening, hydrogenated (Crisco)				478.894			
Soybean oil	70	70	70	20	233.33		
Corn oil						80.8	80.8
Cellulose	50	50	50	50.921	166.67	51.8	51.8
Mineral Mix, w/o Ca & P (98057)	13.388	13.388	13.388	18.2	44.627		
Mineral Mix (S10001)						36.3	36.3
Calcium Carbonate	16.3	17.5	24	26.25	54.33		
Calcium Phopshate, monobasic, monohydrate	21.8	18.7	1.7	28	72.667		
Potassium Phosphate, dibasic							
Vitamin Mix, AIN-93-VX (94047)	15	15	15	20.385	50		
Vitamin Mix (V10001)						10.4	10.4
Choline Bitartrate	2.5	2.5	2.5	3.75	8.33	2.07	2.07
TBHQ, antioxidant	0.014	0.014	0.014	0.1	0.047		
Indole-3-Carbinol (I3C)						2	0
Protein/Amino acid, g/Kg	79.605	174	696	273		211.44	211.44
CHO, g/kg	695.8185	595	36.7787	59		590.5594	590.5594
Fat, g/Kg	70.915	72	78	502		80.8	80.8
Fiber, g/Kg	50	50	50	50.921		51.8	51.8
Ca, g/Kg	10.0045	10	10.0303	15		5.35	5.35
P, g/Kg	6.0033	6	6.0182	9		12.8	12.8
kcal/g	3.74	3.7	3.63	5.8		4.04	4.04

Table S2. List of antibodies used in this study

Target	Antibody	Company	Catalog number	Dilution
α4β7	Rat monoclonal anti-mouse α4β7 Integrin, Brilliant Violet (BV)421 or AF647, clone DATK32	BD Biosciences	566294	1:100
BrdU	Mouse monoclonal anti-BrdU, APC, clone B44 (for tissue IF staining)	BD Pharmingen	51-23619L	1:200
	Mouse monoclonal anti-BrdU (for tissue immunohistochemistry)	Sigma-Aldrich	B2531	1:2000
	Anti-BrdU, FITC (for flow cytometry, from FITC BrdU Flow Kit)	BD Pharmingen	51-23614L	1:70
CD3	Armenian Hamster monoclonal anti-mouse CD3e, FITC clone 2c11	Invitrogen	11-0031-82	1:200
CD4	Rat monoclonal anti-mouse CD4, APC-eFluor 780, clone RM4-5	ThermoFisher Scientific	47-0042-82	1:200
CD5	Rat monoclonal anti-mouse CD5 FITC clone 53-7.3	BioLegend	100606	1:200
CD8α	Rat monoclonal anti-mouse CD8a, PerCP/Cyanine5.5, clone 53-6.7	Biolegend	100734	1:200
CD8β	Rat monoclonal anti-mouse CD8b, FITC clone eBioH35-17.2	Invitrogen	11-0083-85	1:200
CD11b	Rat monoclonal anti-mouse CD11b, Brilliant UltraViolet (BUV)737, clone M1/70	BD Biosciences	612801	1:400
CD11c	Rat monoclonal anti-mouse CD11c, FITC or PE-Cy7, clone N418	ThermoFisher Scientific	11-0114-82	1:200 or 1:300
CD16/32	Anti-CD16/32 Fc block	Invitrogen	14-0161-86	1:400
CD19	Rat monoclonal anti-mouse CD19, FITC, clone eBio1D3	ThermoFisher Scientific	11-0193-82	1:200
CD22	Rat monoclonal anti-mouse CD22, FITC or Alexa Fluor (AF)647, clone OX-97 (for flow cytometry)	Biolegend	126108	1:150 or 1:200
	Goat polyclonal anti-mouse CD22 (for tissue IF staining)	R&D Systems	AF2296	1:100
CD45	Rat monoclonal anti-mouse CD45, BUV395, clone 30-F11	BD Biosciences	564279	1:400
CD45R (B220)	Rat monoclonal anti-mouse B220, biotin clone RA3-6B2	Biolegend	103204	1:400
CD80	Armenian Hamster monoclonal anti-mouse CD80 FITC , clone 16-10A1	Biolegend	104705	1:200
CD90.2	Rat monoclonal anti-mouse CD90.2, BUV737, clone 53-2.1	BD Biosciences	741701	1:300 or 1:400
CD103	Rat monoclonal anti-mouse CD103, BV711 or BV421, clone M290	BDBioscience s	564320	1:300
CD115	Rat monoclonal anti-mouse CD115, biotin, clone AFS98	Invitrogen	13-1152-82	1:400
CD140a (PDGFRA)	Rat monoclonal anti-mouse CD140a biotin, clone APA5 (eBio)	Invitrogen	323504	1:400

CD274 (PD-L1)	Rat monoclonal anti-mouse CD274 PE/Dazzle 594, clone 10F.9G2	Biolegend	124323	1:100 or 1:200
FoxP3	Rat monoclonal anti-mouse FoxP3, PE-Cyanine5.5, clone FJK-16s	ThermoFisher Scientific	35-5773-82	1:100 or 1:200
Gata3	Rat monoclonal anti-mouse Gata-3, PE or AF647, clone TWAJ	ThermoFisher Scientific	12-9966-42	1:100 or 1:200
Gr-1	Rat monoclonal anti-mouse Gr-1, AF488 or FITC, clone RB6-MC5	Biolegend	108417	1:200
IgA	Rat anti-mouse IgA, FITC clone C10-3 or biotin clone C10-1	BD Pharmingen	559354 or 556978	1:200 or 1:400
Ly6C	Rat monoclonal anti-mouse Ly6C, APC, clone HK1.4	ThermoFisher Scientific	17-5932-82	1:200
Ly6G	Rat monoclonal anti-mouse Ly-6G, PerCP-Cy5.5 or biotin, clone 1A8	BD Biosciences	560602	1:200 or 1:300 or 1:400
MBP	Rat monoclonal anti-mouse MBP, clone MT2-14.7.3	Mayo Clinic, Arizona	N/A	1:1000
MHCII (I-A/I-E)	Rat monoclonal anti-mouse I-A/I-E, FITC, APC-eFluor780, BV711, or biotin, clone M5/114.15.2	BD Biosciences	563414	1:300 or 1:400
NK1.1	Mouse monoclonal anti-mouse NK1.1, FITC or biotin, clone PK136	Biolegend	108706	1:200 or 1:400
Rorgt	Mouse monoclonal anti-mouse Rorgt, PE or BV421, clone Q31-378	BD Biosciences	562894	1:100 or 1:200
TCRαβ	Armenian hamster monoclonal anti-mouse TCR beta, PE-Cyanine7, clone H57-597	Biolegend	109222	1:200
TCRγδ	Armenian hamster monoclonal anti-mouse TCRg/d APC, clone eBioGL3	ThermoFisher Scientific	17-5711-82	1:200
	Armenian hamster monoclonal anti-mouse TCRg/d BV421, clone GL3	Biolegend	118120	1:200
Ter-119	Rat monoclonal anti-mouse Ter-119, FITC or biotin, clone Ter-119	Biolegend	116206	1:200 or 1:400

Table S3. List of primers used in this study

Gene	Sequence (5' → 3')	Ref.*
Ada	Forward: TCACCCCTGATGACGTTGTG<br>Reverse: ACCGGACCTTGATGCCAAAT	(1)
Aldh1	Forward: GCAAAGCTGCGGTGCTATG<br>Reverse: TCACACAAGTCACCCCTTCTC	(2)
Aldh1a1	Forward: ATACTTGTCGGATTTAGGAGGCT<br>Reverse: GGGCCTATCTTCCAAATGAACA	(2)
Anpep	Forward: ACGGCCCCATGAAGAGGTAT<br>Reverse: GAGGACGGTTGACCCAGTTG	(3)
Apoa4	Forward: TGGTGTGGGATTACTTTACCCAG<br>Reverse: TTGTCCTGGAAGAGGGTACTGA	
Ccl24	Forward: CTCTGAACCCACAGCAGCTT<br>Reverse: AATTCCAGAAAACCGAGTGG	
Cd38	Forward: GGCCGGAGATGAGAGATCAGA<br>Reverse: GGGGCGTAGTCTTCTCTTGTG	
Cdx1	Forward: GCGGTGGCAGCGGTAAG<br>Reverse: TACCGGCTGTAGTGAAACTCCT	
Egr1	Forward: TGAGCACCTGACCACAGAGTC<br>Reverse: ATAGGTGATGGGAGGCAACCG	
IL17rc	Forward: CGTGGGTTCTGCGGTATTTG<br>Reverse: GGACATTGTCACCATCAGGCA	(1)
Isx	Forward: AGGATCAGCCCCAAGAAGAGAA<br>Reverse: GTCAGGGTAATGGGTGAAGTGG	
Lct	Forward: GTCCTCTTCGCTCTTGTGCT<br>Reverse: GCTGTGTTGTCCCTTGCTTG	
Maf	Forward: CCCCACACATACCCTGGACT<br>Reverse: TTGATGGCCCGGTTCAAAGG	
Nte5	Forward: TCCTGCAAGTGGGTGGAATC<br>Reverse: AGATGGGCACTCGACACTTG	
Oat	Forward: TGGCGGTTTATACCCTGTGTC<br>Reverse: AGTGGGTTTCCGCCGTATG	
Rara	Forward: CTGGGCAAGTACACTACGAACA<br>Reverse: TGCACTTGGTGGAGAGTTCAC	
Rarb	Forward: GAAAACGACGACCCAGCAAG<br>Reverse: CACGTTCGGCACCTTTCG	
Rarg	Forward: TCTGGACATCCTAATGCTGCG<br>Reverse: GCATTGTGCATCTGGGTTCG	
Rbp1	Forward: GTGGATCGAGGGTGATGAACT<br>Reverse: GGTTATCTCCTCGGGCTGTT	
Rbp2	Forward: CCGCAACTACGACCTGGATT<br>Reverse: AGGTGACCAGGGTCTTGACA	
Rdh7	Forward: GTGTCTTTGTGTGGTGGTGGTTAC<br>Reverse: CCACAGCTTCTCTATGCTGTGTGA	(2)
Rpl13	Forward: AAGAAGGGAGACAGTTCTGCTG<br>Reverse: TGTGATAACTCTGGCCTTTTCCTT	(3)
Tgm2	Forward: AGGAGAAGAGCGAAGGGACAT<br>Reverse: TGACCTCGGCAAACACGAAG	
Treh	Forward: GGACTCGTACTGGGTGATGG<br>Reverse: TGGGGATATGTCCGTAGGTCT	

*Reference (this study if blank)

SI References

1. A. González-Loyola, *et al.*, c-MAF coordinates enterocyte zonation and nutrient uptake transcriptional programs. *J Exp Med* **219**, e20212418 (2022).

2. Y. G. Cao, *et al.*, Faecalibaculum rodentium remodels retinoic acid signaling to govern eosinophil-dependent intestinal epithelial homeostasis. *Cell Host Microbe* **30**, 1295-1310.e8 (2022).

3. Z. A. Sullivan, *et al.*, γδ T cells regulate the intestinal response to nutrient sensing. *Science* **371** (2021).
